# Subgenome Partitioning and Polyploid Genome Evolution in the Loach Family Botiidae (Order Cypriniformes)

**DOI:** 10.1002/advs.202505411

**Published:** 2025-07-07

**Authors:** Yunyun Lv, Jia Li, Yanping Li, Yu Huang, Qiang Lai, Zhengyong Wen, Jun Wang, Yang He, Jinrong Shi, Zejin Huang, Ying Jiang, Yves Van de Peer, Qiong Shi, Biwen Xie, Yongming Wang

**Affiliations:** ^1^ Key Laboratory of Sichuan Province for Fishes Conservation and Utilization in the Upper Reaches of the Yangtze River College of Life Science Neijiang Normal University Neijiang 641100 China; ^2^ Laboratory of Aquatic Genomics College of Life Sciences and Oceanography Shenzhen University Shenzhen 518067 China; ^3^ State Key Laboratory of Breeding Biotechnology and Sustainable Aquaculture Institute of Hydrobiology Chinese Academy of Sciences Wuhan 430072 China; ^4^ Zizhong County Fishery Development Center Neijiang 641200 China; ^5^ Department of Plant Biotechnology and Bioinformatics Ghent University Ghent 9052 Belgium; ^6^ Center for Plant Systems Biology Vlaams Instituut voor Biotechnologie Ghent 9052 Belgium; ^7^ Department of Biochemistry Genetics and Microbiology University of Pretoria Pretoria 0028 South Africa; ^8^ College of Horticulture Nanjing Agricultural University Nanjing 210095 China; ^9^ Key Laboratory of Freshwater Fish Reproduction and Development (Ministry of Education) Southwest University School of Life Sciences Chongqing 400715 China

**Keywords:** Cypriniformes, genome evolution, loaches, polyploidy, subgenome partitioning

## Abstract

Vertebrates have undergone two rounds of whole‐genome duplication (WGD), termed 1R and 2R, with a third, teleost‐specific duplication (TSGD or 3R) occurring in ray‐finned fishes. In the order Cypriniformes, additional lineage‐specific WGDs have further contributed to species diversification. While polyploidy is well characterized in species like common carp and goldfish, other polyploid taxa—particularly loaches—remain understudied. Here, high‐quality, chromosome‐level genome assemblies are presented for two loach species: *Sinibotia superciliaris* (Golden Chinese Loach) and *Parabotia fasciatus* (Yichang Sand Loach). By integrating these genomes into a comparative framework with 20 other cypriniform species, key phylogenetic relationships are reconstructed, and introduce a novel subgenome partitioning method (M3). Unlike previous approaches, M3 uses differential sequence divergence to accurately and rapidly assign subgenomes, completing partitioning within minutes and outperforming existing tools. Applying M3, a markedly reduced subgenome is uncovered in the Golden Chinese Loach, with lineage‐specific molecular changes in several candidate genes, suggesting potential adaptive significance. This study offers a comprehensive view of polyploidy and subgenome evolution in loaches, highlighting the genomic complexity shaped by repeated WGDs in *Cypriniformes* and providing valuable resources for future research on vertebrate genome evolution and adaptation.

## Graphical abstract

This study investigates subgenome evolution and polyploidy in Cypriniformes fish, revealing complex phylogenetic relationships and genomic dynamics. By applying advanced subgenome partitioning methods, it uncovers novel insights into transposon activity and gene fractionation. The findings deepen understanding of vertebrate genome evolution and highlight challenges in reconstructing polyploid lineages, inviting further exploration in evolutionary biology.

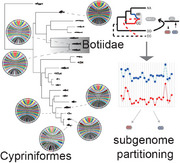

## 1 Introduction

All vertebrates share two ancient whole‐genome duplications (WGDs), commonly known as 1R and 2R,^[^ ^1^, ^2^, ^3^, ^4^ ^]^ while ray‐finned fishes experienced an additional WGD, referred to as the ‘teleost‐specific’ genome duplication (TSGD) or 3R.^[^ ^5^, ^6^, ^7^ ^]^ WGD is a major evolutionary driving force that has played a crucial role in the evolution of vertebrates.^[^ ^8^, ^9^ ^]^ Understanding the occurrence, mechanisms, and consequences of these WGD events is fundamental to evolutionary biology.^[^ ^9^, ^10^ ^]^ These ancient duplication events include the first and second rounds (1R and 2R), which occurred ≈400 million years ago at the base of vertebrate evolution,^[^ ^3^ ^]^ and the third round (3R), estimated to have occurred ≈320 million years ago in an ancestor of teleost fishes.^[^ ^11^, ^12^ ^]^ In addition to these events, certain ray‐finned fish lineages have undergone further lineage‐specific WGDs.^[^ ^5^ ^]^ Notable examples include species within the order Cypriniformes, such as the common carp (*Cyprinus carpio*)^[^ ^13^, ^14^ ^]^ and goldfish (*Carassius auratus*),^[^ ^15^ ^]^ as well as species in the order Salmoniformes (e.g., Atlantic salmon),^[^ ^16^, ^17^ ^]^ and Acipenseriformes, including starlet sturgeon,^[^ ^18^ ^]^ paddlefish,^[^ ^19^ ^]^ and Chinese sturgeon.^[^ ^20^ ^]^

Previous studies have revealed that three families within Cypriniformes, such as Catostomidae (suckers), Botiidae (loaches), and Cyprinidae (carps and minnows), underwent independent polyploidization events.^[^ ^21^ ^]^ Moreover, at least 13 independent WGD events have been identified within the subfamily Cyprininae. These events include allopolyploid and autopolyploid origins; for example, the lineages *Schizothoracinae* and *Schizopygopsinae* are believed to result from autopolyploidy.^[^ ^21^ ^]^ Due to the frequency and diversity of WGD events, Cypriniformes serves as an excellent model for studying genome duplication and polyploid evolution. However, the complex and reticulate evolutionary history of these lineages poses significant challenges for accurately analyzing and interpreting polyploid genomes.

Research on WGDs in Cypriniformes has primarily concentrated on species, such as the common carp and goldfish, which have been extensively investigated for their evolutionary relationships and genome‐level characteristics, including subgenome partitioning, subgenome asymmetry, and functional divergence.^[^ ^13^, ^14^, ^15^, ^22^, ^23^ ^]^ In addition, one study explored the genome evolution of other Cyprinidae species, incorporating genomes from allopolyploid lineages, such as *Spinibarbus*, *Luciobarbus*, and *Procypris*.^[^ ^24^ ^]^ These analyses have further clarified that the subgenome dominance patterns observed in these polyploids are likely shaped by a combination of maternal genome dominance and differential densities of transposable elements across subgenomes.^[^ ^24^ ^]^

In contrast, other polyploid species within the order Cypriniformes, such as loaches, have received comparatively less attention. Although genomic studies on Botiidae species have been conducted,^[^ ^25^, ^26^ ^]^ a comprehensive evolutionary analysis of polyploid loaches is lacking. Key questions remain unresolved at the whole‐genome level, including whether the WGDs in loaches represent cases of allopolyploidy or autopolyploidy, the timing of these events, and whether they coincide with critical periods in evolutionary history, although some gene‐based analyses have attempted to estimate divergence times.^[^ ^27^ ^]^

In the current study, we present newly assembled, chromosome‐level genomes for two loach species: *Sinibotia superciliaris* (Golden Chinese Loach) and *Parabotia fasciatus* (Yichang Sand Loach) and introduce a novel subgenome partitioning approach. By integrating these high‐quality genomes with data from 18 previously sequenced species in the order Cypriniformes, we performed a comprehensive comparative genomic analysis, with a particular focus on the evolution of polyploidy in the loach family Botiidae. Unless otherwise specified, references to polyploid loaches in this study pertain specifically to polyploid members of Botiidae, rather than to polyploid species in other loach families such as Cobitidae. Genome‐scale phylogenetic reconstruction, followed by subgenome reassembly, enabled us to resolve complex evolutionary relationships within Cypriniformes.^[^ ^25^, ^28^ ^]^ Through comparative subgenomic analyses, we elucidated the mechanisms underlying polyploid formation and the post‐hybridization evolution of subgenomes in Botiidae loaches. Taken together, this study integrates genomic data from 20 cypriniform species across eight families, providing a comprehensive framework for understanding the origin and evolution of polyploidy in Cypriniformes, with a particular focus on the family Botiidae.

## 2 Results and Discussion

### 2.1 High‐Quality Chromosome‐Level Genome Assembly and Annotation of *Sinibotia superciliaris* (Golden Chinese Loach) and *Parabotia fasciatus* (Yichang Sand Loach)

We generated ≈128 Gb of sequencing data for the *S. superciliaris*, comprising ≈68 Gb of long‐read and ≈60 Gb of short‐read data (Tables S1 and S2, Supporting Information). We generated ≈120 Gb of sequencing data for *P. fasciatus*, comprising ≈59 Gb of long‐read and ≈61 Gb of short‐read data (Tables S3 and S4, Supporting Information). After correcting the errors and assembly, we obtained high‐continuity draft genomes for both species. The genome size of the *S. superciliaris* was estimated to be 807 Mb, with a scaffold N50 length of ≈15.5 Mb (Table S5, Supporting Information). The genome size of *P. fasciatus* was 614 Mb, with a scaffold N50 length of 22 Mb (Table S6, Supporting Information).

Using high‐throughput chromosome conformation capture (Hi‐C) data, we assembled chromosome‐level genomes, resulting in 49 and 25 clearly defined clusters for *S. superciliaris* and *P. fasciatus*, respectively, representing their chromosome sequences (Figures S1 and S2, Supporting Information). Genome completeness, as assessed using the Benchmarking Universal Single‐Copy Orthologue (BUSCO) tool, reached 97.6% in both species (Tables S7 and S8, Supporting Information). However, the proportion of complete and duplicated BUSCOs (D) was significantly higher in Golden Chinese Loach (31.60%) than in *P. fasciatus* (1.60%), reflecting the polyploid nature of the Golden Chinese Loach.

Annotation of repetitive sequences revealed that repetitive elements accounted for 32.32 and 40.04% of the genomes in the *S. superciliaris* and *P. fasciatus*, respectively (Tables S9 and S10, Supporting Information). Gene annotation identified 42 157 and 26 391 protein‐coding genes for *S. superciliaris* and *P. fasciatus*, respectively. BUSCO analysis of the annotated protein‐coding genes indicated completeness levels of 94.7% for the Golden Chinese Loach and 91.6% for the Yichang Sand Loach (Tables S11 and S12, Supporting Information).

### 2.2 Genome‐Level Species Evolution in Cypriniformes

**Figure 1:**
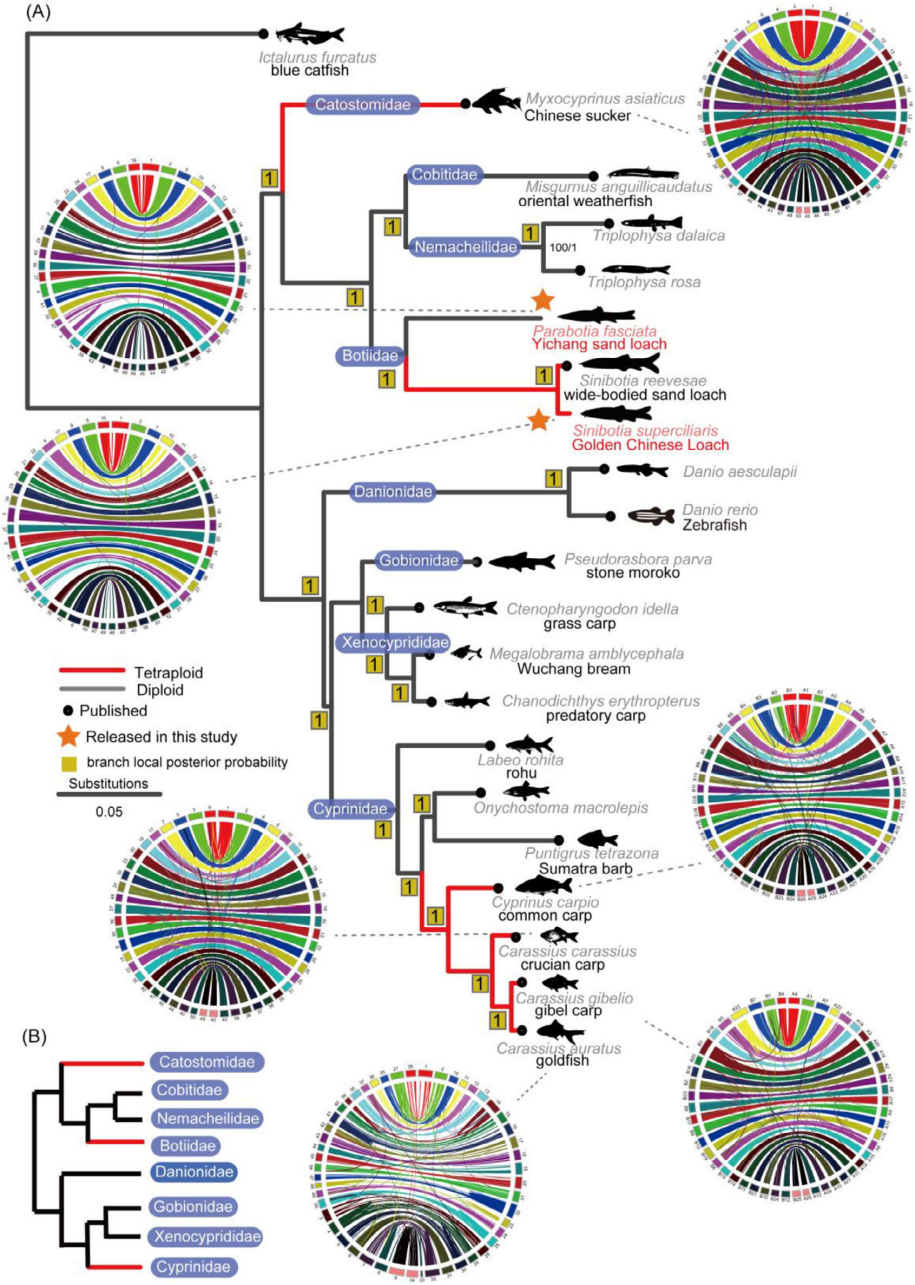
Evolutionary relationships within *Cypriniformes* and the origin of polyploid loaches. A) Phylogenetic tree of *cypriniform* species reconstructed using a coalescent‐based method. Red branches represent polyploid species, while gray branches indicate diploid species. Branch lengths correspond to the number of accumulated nucleotide substitutions. Numbers next to the nodes denote local posterior probabilities. Brown stars highlight the two newly assembled genomes reported in this study for Yichang Sand Loach and Golden Chinese Loach. B) Hierarchical clustering of *cypriniform* families based on the relationships summarized in panel A. The *Danionidae* family is shown with a dark blue background to indicate a topological inconsistency between concatenation‐based and coalescent‐based methods. The coalescent‐based topology is shown here, while the concatenation‐based result is presented in Figure S3 (Supporting Information).

We integrated the genomes of 20 species from the order Cypriniformes for comparative genomics analysis. The dataset included two newly assembled genomes presented in this study (the *S. superciliaris* and the *P. fasciatus*, marked with brown stars in **Figure** **1**A) as well as previously assembled genome of *S. reevesae* (wide‐bodied loach),^[^ ^29^ ^]^ and 17 additional chromosome‐level genomes from members of Cypriniformes. All 17 previously published genomes feature high‐quality gene structure annotations and are publicly available from the National Center for Biotechnology Information, with the accession numbers listed in Table S13 (Supporting Information). We selected the blue catfish (*Ictalurus furcatus*) from the order Siluriformes as an outgroup for the comparative analysis (Table S13, Supporting Information).

Through gene family clustering, we identified 771 single‐copy gene families across all genomes. These single‐copy genes were aligned and concatenated into a supermatrix of 1 284 987 bp for phylogenetic analysis. We employed a concatenation‐based approach to infer the species tree. Phylogenetic trees constructed using the maximum likelihood (ML) and Bayesian inference (BI) methods (see Experimental Section) yielded highly consistent topologies, with strong node support across the tree, providing a robust evolutionary framework for species within the order Cypriniformes (Figure S3, Supporting Information). The resulting topology at the family level was: (outgroup, ((Cobitidae, Nemacheilidae), Botiidae), Catostomidae), (((Gobionidae, Xenocyprididae), (Danionidae, Cyprinidae))) (Figure S3, Supporting Information).

We also reconstructed phylogenies for each of the 771 single‐copy gene families individually and used a coalescent‐based method to infer a consensus species tree (Figure 1A). Notably, the topology obtained from this approach differed from that of the concatenation‐based tree. The coalescent‐based topology was: (outgroup, ((Cobitidae, Nemacheilidae), Botiidae), Catostomidae), (Danionidae, ((Gobionidae, Xenocyprididae), Cyprinidae)) (Figure 1B).

The discordance between the concatenation‐based and coalescent‐based trees mainly involved the phylogenetic position of Danionidae. This inconsistency was likely attributable to incomplete lineage sorting (ILS), potentially resulting from a rapid radiation event during the early diversification of Danionidae. Such phylogenetic conflict caused by ILS was observed in a previous study,^[^ ^30^ ^]^ particularly in lineages undergoing rapid speciation.

In addition to the phylogenetic analysis, we compared the chromosome sequences of each species and identified seven species that have undergone WGD events. These species included the Chinese sucker (*Myxocyprinus asiaticus*), wide‐bodied sand loach (*Sinibotia reevesae*), the Golden Chinese Loach, common carp, crucian carp (*Carassius carassius*), gibel carp (*Carassius gibelio*), and goldfish. The genomes analyzed exhibited a clear one‐to‐one correspondence between chromosomes, as shown in the circos plots in Figure 1A. This pattern strongly suggests that a WGD event occurred during their evolutionary history. Despite this overall consistency, some minor chromosomal rearrangements were present, indicating post‐duplication genome reorganization.

As revealed by the phylogenetic tree, the seven WGD events identified in Cypriniformes do not share a monophyletic origin but are distributed across three distinct families of Catostomidae (suckers), Botiidae (loaches), and Cyprinidae (carps). This finding is consistent with previous studies.^[^ ^21^ ^]^ However, due to limitations in genome quality and gene annotation in earlier datasets,^[^ ^24^, ^26^ ^]^ reports of additional autopolyploid and allopolyploid WGD events in Cyprinidae (particularly in carps) were not fully recovered in our analysis. Future studies incorporating more high‐quality genomes from across Cypriniformes will be essential to resolve the patterns and origins of polyploidy within this diverse order.

In addition, diploid cypriniform species that have undergone WGD typically exhibit a haploid chromosome number of 25, while tetraploid species have a haploid chromosome number of ≈50. This pattern suggests that 25 represents the ancestral haploid chromosome number for species within Cypriniformes.

### 2.3 Allopolyploid Origin of Golden Chinese Loach and Wide‐bodied Sand Loach

**Figure 2:**
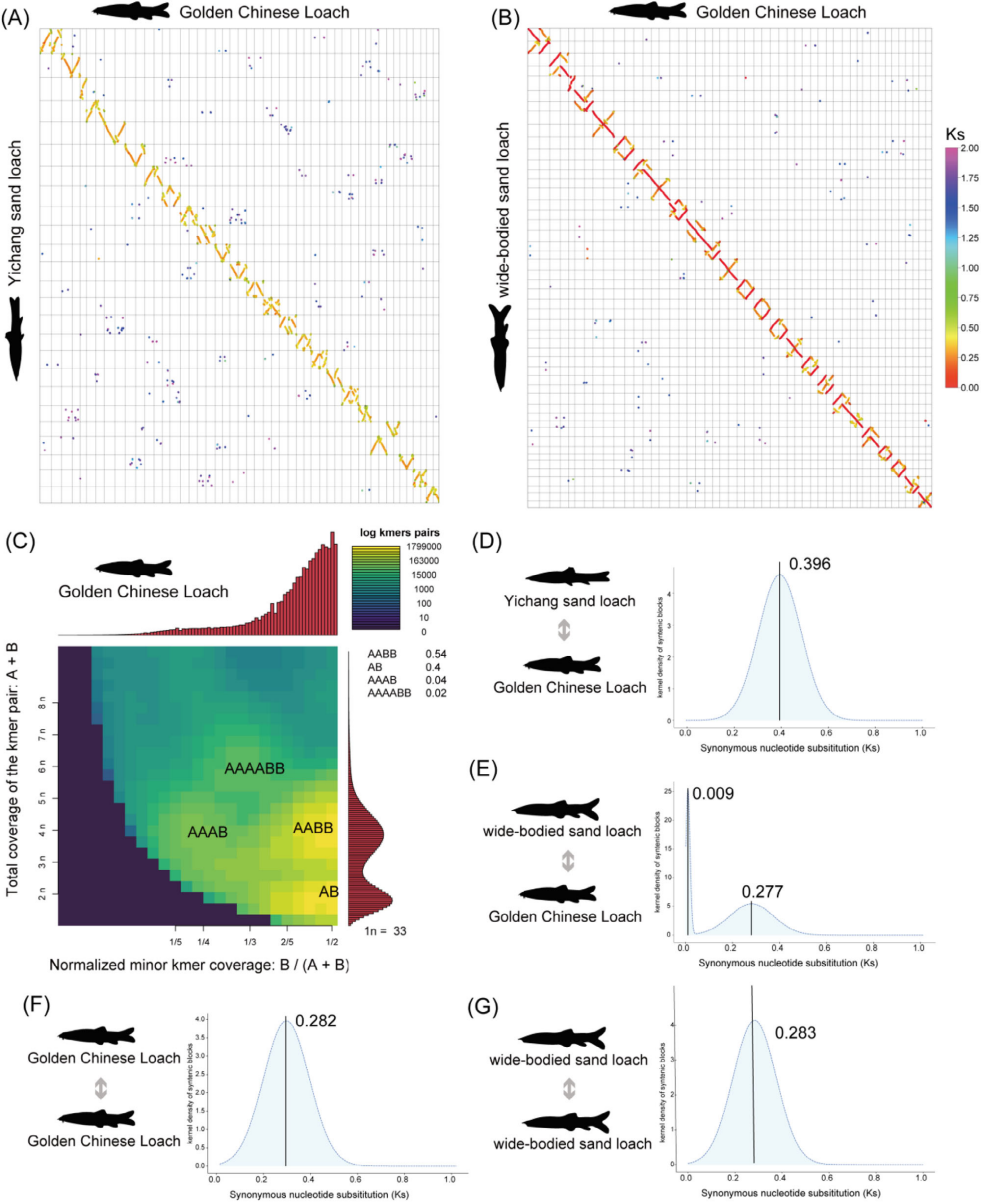
Identification of allopolyploidy in loaches. A) Dot plot showing conserved syntenic blocks and the distribution of synonymous substitution rates (*Ks*) between the Yichang Sand Loach (*P. fasciatus*) and the Golden Chinese Loach(*S. superciliaris*). B) Dot plot and *Ks* distribution for the syntenic genes between the wide‐bodied sand loach (*S. reevesae*) and the Golden Chinese Loach. One chromosome from the wide‐bodied sand loach showed strong collinearity with two distinct chromosomes in the Golden Chinese Loach. The *Ks* values for gene pairs on one chromosome were notably higher (darker color) than those on the other, indicating asymmetric divergence and supporting an allopolyploid origin. C) Heterozygous k‐mer pair analysis confirmed that Golden Chinese Loach is an allotetraploid, with the AABB configuration being the most prevalent. D) Unimodal *Ks* distribution for the syntenic genes between Yichang Sand Loach and Golden Chinese Loach, with the corresponding *Ks* value. E) Bimodal *Ks* distribution for the syntenic genes between wide‐bodied sand loach and Golden Chinese Loach, with corresponding *Ks* values for each peak. F) Unimodal *Ks* distribution for the syntenic genes within the Golden Chinese Loach genome and the corresponding *Ks* value. G) Unimodal *Ks* distribution for the syntenic genes within the wide‐bodied sand loach genome and the corresponding *Ks* value.

To investigate the polyploid origin of the wide‐bodied sand loach and the Golden Chinese Loach genomes, we computed the genome‐wide synonymous substitution rates (Ks) of the homologous gene pairs between the two species and the closely related diploid Yichang Sand Loach (**Figure** **2**A). Then, we compared the genome‐wide Ks values between the wide‐bodied sand loach and the Golden Chinese Loach (Figure 2B). Additionally, heterozygous k‐mer pair analysis revealed that a majority of genotypes in the Golden Chinese Loach genome (54%) exhibited an AABB configuration, with 40% showing an AB genotype (Figure 2C), suggesting widespread fixed heterozygosity consistent with an allotetraploid origin.

The *Ks* distribution of the syntenic gene pairs between the Yichang Sand Loach and the Golden Chinese Loach revealed a unimodal peak centered at 0.396 (Figure 2D). In contrast, the *Ks* distribution of syntenic gene pairs between the wide‐bodied sand loach and the Golden Chinese Loach displayed a bimodal pattern, with peaks at 0.009 and 0.277 (Figure 2E). Furthermore, the intra‐genomic *Ks* distributions within the Golden Chinese Loach and the wide‐bodied sand loach were unimodal, with peaks at 0.282 and 0.283, respectively (Figure 2F,G), indicative of a shared WGD event.

Together, these *Ks* patterns demonstrate that divergence between the Yichang Sand Loach and Golden Chinese Loach (*Ks* peak at 0.396) predated the WGD event shared by the Golden Chinese Loach and wide‐bodied sand loach (*Ks* peaks at 0.28). This temporal sequence, combined with the k‐mer genotype analysis, strongly supported the hypothesis that the Golden Chinese Loach and wide‐bodied sand loach have an allopolyploid (i.e., hybrid tetraploid) origin, whereas the Yichang Sand Loach remains diploid. The *Ks* peak at 0.009 represents the recent divergence between the Golden Chinese Loach and the wide‐bodied sand loach, further supporting their close relationship as independently formed lineages derived from an allopolyploid ancestor.

Previous fragmentary evidence has pointed toward an allopolyploid origin for species in the subfamily Botiinae (loaches). For example, recent studies on four genera and five species of polyploid botiids have suggested allopolyploidy based on both gene phylogenetic analyses and genome‐wide k‐mer surveys.^[^ ^27^ ^]^ Our present genome‐wide investigation provides the first comprehensive genomic confirmation of the allopolyploid origin of loaches, reinforcing the idea that allopolyploidy may represent a predominant mode of polyploidization within Cypriniformes.

### 2.4 Subgenome Partitioning of Allotetraploid Genomes

**Figure 3:**
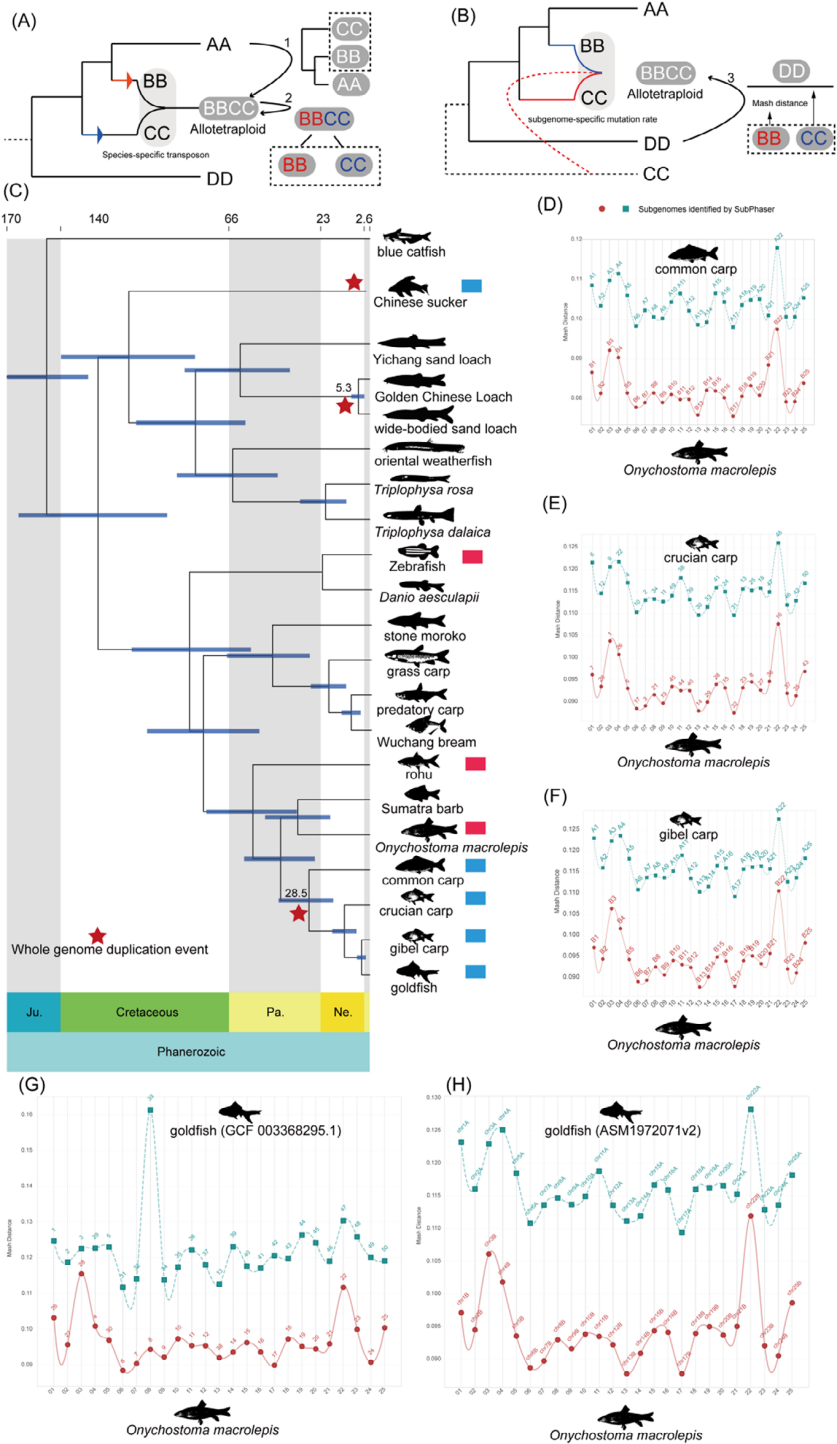
Methods for identifying allopolyploid subgenomes. A) Diagram illustrating two commonly used strategies for identifying subgenomes based on comparisons with ancestral diploid species and transposon insertion patterns, corresponding to methods M1 and M2 described in the main text. Blue and red colors represent different subgenomes but do not indicate parental (maternal/paternal) origin. B) A novel approach (method M3) proposed in this study for subgenome partitioning is based on differential sequence divergence accumulated due to varying ancestral divergence times among the subgenomes. In this model, species DD served as a diploid reference, while BB and CC represented the ancestral progenitors of an allotetraploid. However, this method does not distinguish between maternal and paternal progenitors. C) Estimated divergence times of polyploid species within the order Cypriniformes. D–H) Subgenome identification results in different carp species using *Onychostoma macrolepis* as a diploid reference genome. Results are compared with those generated using SubPhaser software.

The separation of subgenomes in the allopolyploid genomes of cypriniform species has been extensively studied,^[^ ^14^, ^15^, ^22^, ^23^ ^]^ with the most common approach comparing chromosomes to the chromosomes of different sister species, to determine whether the chromosomes are similar to those of a sister species, which could potentially identify a progenitor. When successful, this method allows separating the subgenomes (Method 1, hereafter defined as M1 in **Figure** **3**A). An alternative method is based on transposons and separates the subgenomes by exploiting differences in transposon abundance in the different subgenomes. This is achieved through statistical analysis of transposon content or the repeat frequency of short fragments,^[^ ^31^ ^]^ which helps distinguish the allopolyploid regions (Method 2, hereafter defined as M2 in Figure 3A), such as shown for hexaploid Prussian carp.^[^ ^32^ ^]^

Previous studies using the M1 method for subgenome partitioning have focused on carp species, such as the common carp and gibel carp.^[^ ^14^, ^15^ ^]^ As the most recent common ancestor of these species cannot be directly identified on a phylogenetic tree, we applied this method to partition the subgenomes of seven cypriniform species, including Chinese sucker, wide‐bodied sand loach, Golden Chinese loach, common carp, crucian carp, gibel carp, and goldfish. Our results showed that M2 successfully separated the subgenomes in five of these species, including Chinese sucker (Figure S4, Supporting Information), common carp (Figure S5, Supporting Information), crucian carp (Figure S5, Supporting Information), gibel carp (Figure S5, Supporting Information), and goldfish (for both genome assemblies: GCF_003368295.1 in Figure S5 (Supporting Information) and ASM1972071v2 in Figure S6, Supporting Information) into two distinct subgenomes. The results for common carp and gibel carp were consistent with those obtained using the M1 method,^[^ ^14^, ^15^ ^]^ confirming the efficacy of the M2 approach. However, the M2 method did not apply to wide‐bodied sand loach or Golden Chinese Loach possibly because of the relatively low transposon content (≈30%) in the genomes of these species, whereas most teleost genomes contain more than 40% transposable elements. Thus, we compared the transposon content across homologous chromosomes between the wide‐bodied sand loach and the Golden Chinese Loach. The results revealed no differences in transposon content between the corresponding chromosomes (Figures S7 and S8, Supporting Information), suggesting that the two species share a similar evolutionary history of transposon activity during allopolyploid formation, and also highlights a potential limitation of the M2 method, which relies on a statistical analysis of transposon content for subgenome partitioning.

To overcome these limitations, we developed a new subgenome partitioning approach, called Method 3 (M3). This method is based on the premise that the two progenitor diploid species accumulated mutations at different rates before hybridization and formation of an allopolyploid. M3 accounts for the possibility that the two subgenomes experienced asymmetric rates of degeneration following allopolyploidization. In either scenario, the divergence times or post‐polyploidization evolutionary trajectories of the ancestral subgenomes are assumed to differ, which is referred to as the subgenome‐specific mutation rate (Figure 3B). As a result, when chromosomes are compared across closely related species, the two subgenomes display varying levels of sequence differentiation. These differences can be quantified using the Mash distance,^[^ ^33^ ^]^ a measure of genomic dissimilarity. In addition, M3 is compatible with scenarios typically addressed by M1. For example, in cases where the phylogenetic proximity of the outgroup species to one of the subgenomes is unknown, such as when species CC may be derived from ancestral species DD and BB, M3 effectively partitions the subgenomes without requiring previous knowledge of progenitor relationships (Figure 3B).

Unlike M1 and M2, M3 does not require a diploid reference genome from the subgenome progenitors, as in M1, nor does it rely on assessing differences in transposon abundance between subgenomes, as in M2. Instead, M3 only requires homologous reference genomes from related species. We have developed a dedicated pipeline (https://github.com/lvyunyunSCI/Div_m3.git) to facilitate application and reproducibility. We tested this method on several species discussed in this study, and it completed the subgenome partitioning within minutes.

To evaluate the effectiveness of the M3 method, we first applied it to five species, including the Chinese sucker, common carp, crucian carp, gibel carp, and goldfish (indicated by blue rectangles in Figure 3C). Three outgroup species, *Onychostoma macrolepis*, zebrafish, and rohu (*Labeo rohita*), were used as reference genomes. The M3 method performed well for common carp, crucian carp, gibel carp, and goldfish, clearly distinguishing the chromosomal composition of their subgenomes. A lower Mash distance indicated less accumulated variation and thus a closer relationship to the ancestral state.

**Figure 4:**
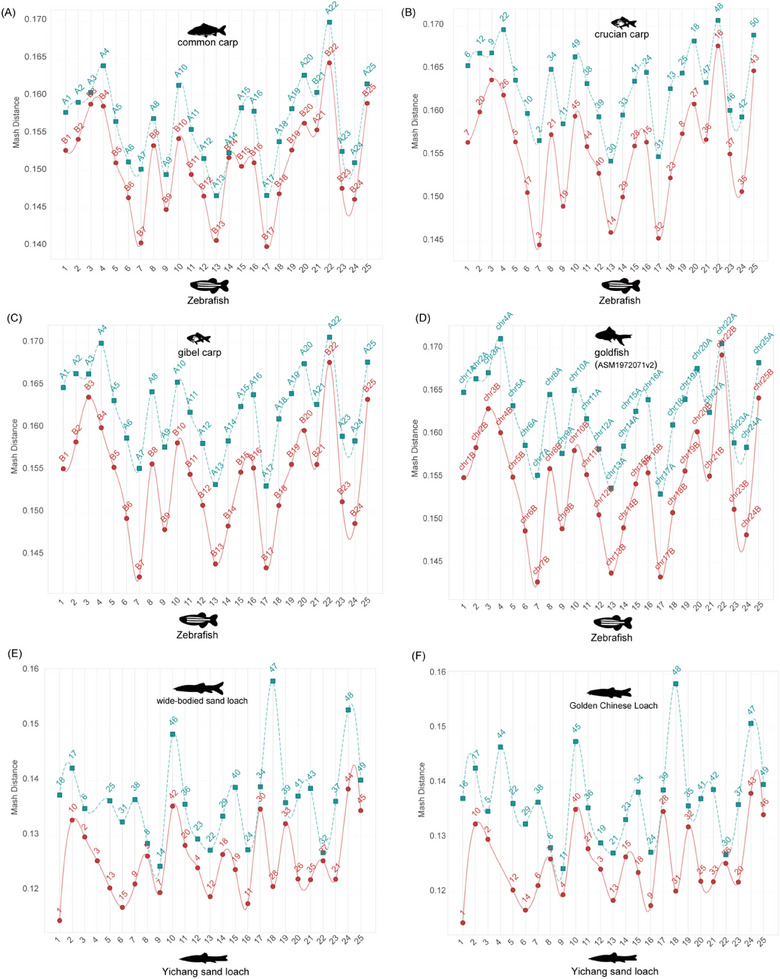
Subgenome partitioning of four carp species and two allopolyploid loaches. A–D) Subgenome partitioning results for four carp species using zebrafish as a reference genome. E) Subgenome partitioning of the wide‐bodied sand loach using the Yichang Sand Loach as the reference genome. F) Subgenome partitioning of the Golden Chinese Loach using the Yichang Sand Loach as the reference genome.

When using *O. macrolepis* as the reference genome, M3 yielded results highly consistent with M2 for common carp (Figure 3D), crucian carp (Figure 3E), gibel carp (Figure 3F), and goldfish (Figure 3G,H). To further test the robustness of M3, we used zebrafish as the reference. Despite its greater phylogenetic distance from the target species compared to *O. macrolepis* (Figure 3C), M3 correctly partitioned the subgenomes of common carp (**Figure** **4**A), crucian carp (Figure 4B), gibel carp (Figure 4C), and goldfish (Figure 4D). Then, we repeated the analysis using rohu as the reference genome and obtained consistent results, accurately identifying the subgenomes in common carp (Figure S9, Supporting Information), crucian carp (Figure S10, Supporting Information), gibel carp (Figure S11, Supporting Information), and goldfish (Figure S12, Supporting Information). These results demonstrate the robustness of M3 across different reference genomes.

Building on these results, we applied the M3 method to wide‐bodied sand loach (Figure 4E) and Golden Chinese Loach (Figure 4F), successfully partitioning their genomes into two subgenomes. In both species, one subgenome consistently showed greater Mash distances, while the other had lower values. Thus, M3 effectively enabled subgenome partitioning in these two loach species. However, M3 failed to differentiate the Chinese sucker subgenomes, indicating that this species falls outside the suitable application range of M3. In contrast, M2 (Figure S4, Supporting Information) performed better for subgenome partitioning of Chinese sucker.

Subgenome partitioning in polyploid genomes, particularly in allopolyploid genomes, is crucial for understanding the distinct evolutionary trajectories of subgenomes following WGD.^[^ ^34^ ^]^ Reliable and effective tools to accurately discriminate subgenomes are essential for deciphering the origins and consequences of polyploid formation.

Although several subgenome partitioning methods have been proposed (e.g., M1 and M2),^[^ ^34^ ^]^ they are not universally applicable. The M1 method requires a diploid progenitor genome closely related to one of the subgenomes.^[^ ^34^ ^]^ However, for polyploid lineages with monophyletic origins, such as the Golden Chinese Loach and wide‐bodied sand loach,^[^ ^35^ ^]^ one diploid ancestor may be extinct, making M1 infeasible. The M2 method is more flexible because it does not require an external reference genome but assumes substantial divergence in transposon content between subgenomes.^[^ ^34^ ^]^ This assumption fails in species with relatively low repeat content, such as the Golden Chinese Loach and wide‐bodied sand loach.

To further evaluate whether repetitive sequences influence the performance of M3 in Golden Chinese Loach, we compared the subgenome partitioning results using masked and unmasked versions of the genome (Figure S13, Supporting Information). The results were identical, indicating that M3 is relatively insensitive to repetitive sequences. Additionally, we analyzed the divergence distributions of transposable elements across the two subgenomes in wide‐bodied sand loach (Figure S14, Supporting Information) and Golden Chinese Loach (Figure S15, Supporting Information). Similar patterns were observed, suggesting that the subgenomes in both species may share similar evolutionary dynamics of transposable elements, thereby limiting the applicability of M2.

In summary, we present M3 as a robust and user‐friendly method for subgenome partitioning, capable of producing accurate results within minutes without requiring diploid progenitor genomes. However, as with any analytical approach, M3 has certain limitations. First, because it relies on differences in Mash distances between each subgenome and selected diploid reference genomes, cases where these distances are very similar may yield ambiguous results. In such scenarios, it is advisable to test multiple reference species to enhance resolution. Second, Mash distance is a probabilistic estimate based solely on k‐mer similarity, which does not account for structural features such as gene order, chromosome architecture, or sequence length. Consequently, complex evolutionary events like homoeologous exchanges or segmental allopolyploidy may reduce its discriminatory power. Lastly, the accuracy of Mash distance calculations is influenced by the choice of k‐mer size and sketch size; inappropriate parameter selection could affect subgenome assignment accuracy.

Despite these limitations, M3 remains a practical and scalable tool for dissecting subgenome structures in polyploid genomes. Its speed, ease of implementation, and independence from progenitor genome information make it particularly valuable for studying polyploid genome evolution in non‐model species.

### 2.5 Evolutionary Relationships and Accumulation of Mutations in *Cypriniformes* after Subgenome Partitioning

Using different subgenome partitioning methods (M2 and M3), we divided the genomes of seven polyploid species within the order Cypriniformes, such as Chinese sucker, wide‐bodied sand loach, Golden Chinese Loach, common carp, crucian carp, gibel carp, and goldfish, into two subgenomes each. To facilitate the comparative genomics analysis, we also included diploid species and selected the blue catfish (*Ictalurus furcatus*) as the outgroup. We conducted comprehensive gene alignment and gene family clustering, identifying 1781 single‐copy genes. These genes were concatenated into a supergene of 3 319 950 base pairs, which served as the basis for phylogenetic reconstruction using the ML and BI approaches.

**Figure 5:**
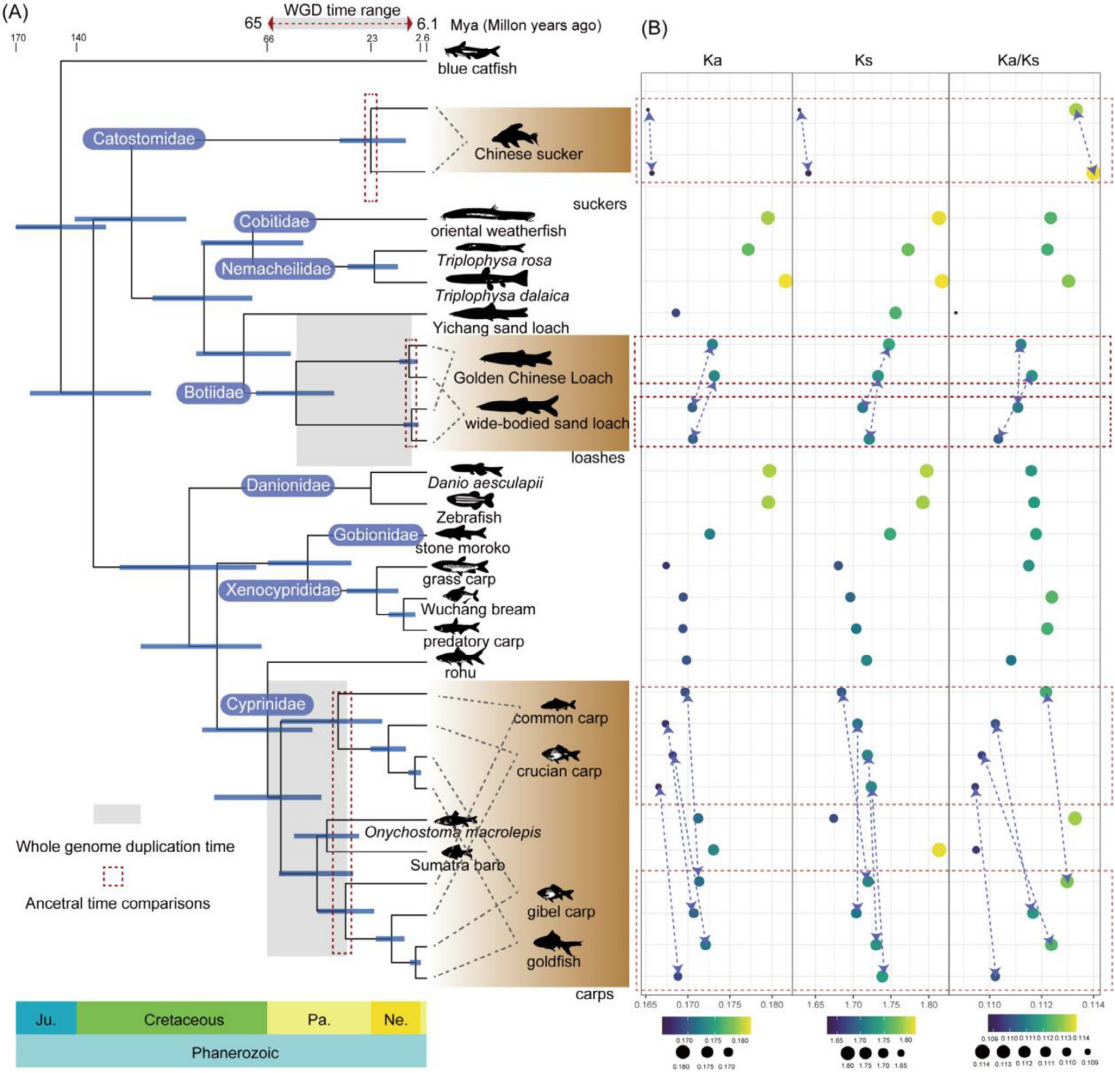
Evolutionary history of Cypriniformes and the subgenome characteristics based on subgenome partitioning. A) A robust phylogenetic relationship of species within the order Cypriniformes reconstructed using subgenomes from polyploid species. Divergence times for the subgenomes and their closest diploid ancestors suggest that polyploidization in Cypriniformes occurred between 65 and 6.1 million years ago (Mya). B) Comparative analysis of *Ka*, *Ks*, and *Ka/Ks* values for diploid and polyploid species in the order Cypriniformes, using outgroup species as references. Although slight differences in *Ka*, *Ks*, and *Ka/Ks* values were observed among the carp species subgenomes, no clear preference for specific subgenomes was detected in loaches or suckers.

Additionally, we constructed a phylogenetic tree for each single‐copy gene family using a coalescent‐based method. The resulting phylogenies from the concatenation‐ and coalescent‐based approaches were fully congruent (**Figure** **5**A), with robust support for all nodes. Notably, the topology at the family level (e.g., Danionidae) was more consistent with previous coalescent‐based trees built without subgenome partitioning, whereas the concatenation‐based trees showed discrepancies. These results suggest that subgenome‐aware phylogenetic reconstruction improves the stability and accuracy of the tree.

To further explore the evolutionary history of polyploidy in Cypriniformes, we estimated the divergence times between subgenomes and their closest diploid relatives. The results indicated that polyploids formed in the three major lineages between ≈60 and 9 million years ago (Figure 5A). Chromosome collinearity analyses revealed that chromosomal evolution across Cypriniformes is generally conserved, with most species maintaining 25 chromosomes and exhibiting few interchromosomal rearrangements (Figure S16, Supporting Information). Exceptions include large‐scale chromosomal fusions in species, such as grass carp (*Ctenopharyngodon idella*), Wuchang bream (*Megalobrama amblycephala*), and predatory carp (*Chanodichthys erythropterus*), which led to a reduction in chromosome number by one (Figure S16, Supporting Information), consistent with previous findings.^[^ ^36^, ^37^, ^38^ ^]^

Many cypriniform species are distributed in regions surrounding the Qinghai‐Tibet Plateau, a geologically dynamic area.^[^ ^39^ ^]^ The rapid uplift of the plateau, driven by the collision of the Eurasian and Indian plates, is known to have promoted species diversification.^[^ ^40^, ^41^ ^]^ Rearrangements of the river systems and river capture likely facilitated cycles of population isolation, divergence, and secondary contact,^[^ ^42^, ^43^ ^]^ conditions that promoted the formation of allopolyploids. The relative karyotypic conservation observed in Cypriniformes (Figure S16, Supporting Information) may have reduced the incompatibilities between subgenomes, further supporting the establishment and stability of polyploids.^[^ ^44^ ^]^

Following subgenome partitioning, we estimated non‐synonymous (*Ka*) and synonymous (*Ks*) substitution rates and their ratio (*Ka/Ks*) across different subgenomes and diploid species, using the blue catfish as a reference. Comparative analyses revealed minor differences in selective pressure between the subgenomes in carps (Figure 5B), but no significant divergence in selective pressure between subgenomes in loaches or the Chinese sucker. Small differences in *Ka* and *Ks* between the subgenomes in loaches and carps suggest varying evolutionary timescales among their hybrid ancestors, leading to different levels of accumulated mutations. These findings agree well with the conceptual framework of the subgenome partitioning approach proposed in this study, particularly the M3 method.

### 2.6 Positively Selected Genes in Size‐reduced Genomes

**Figure 6:**
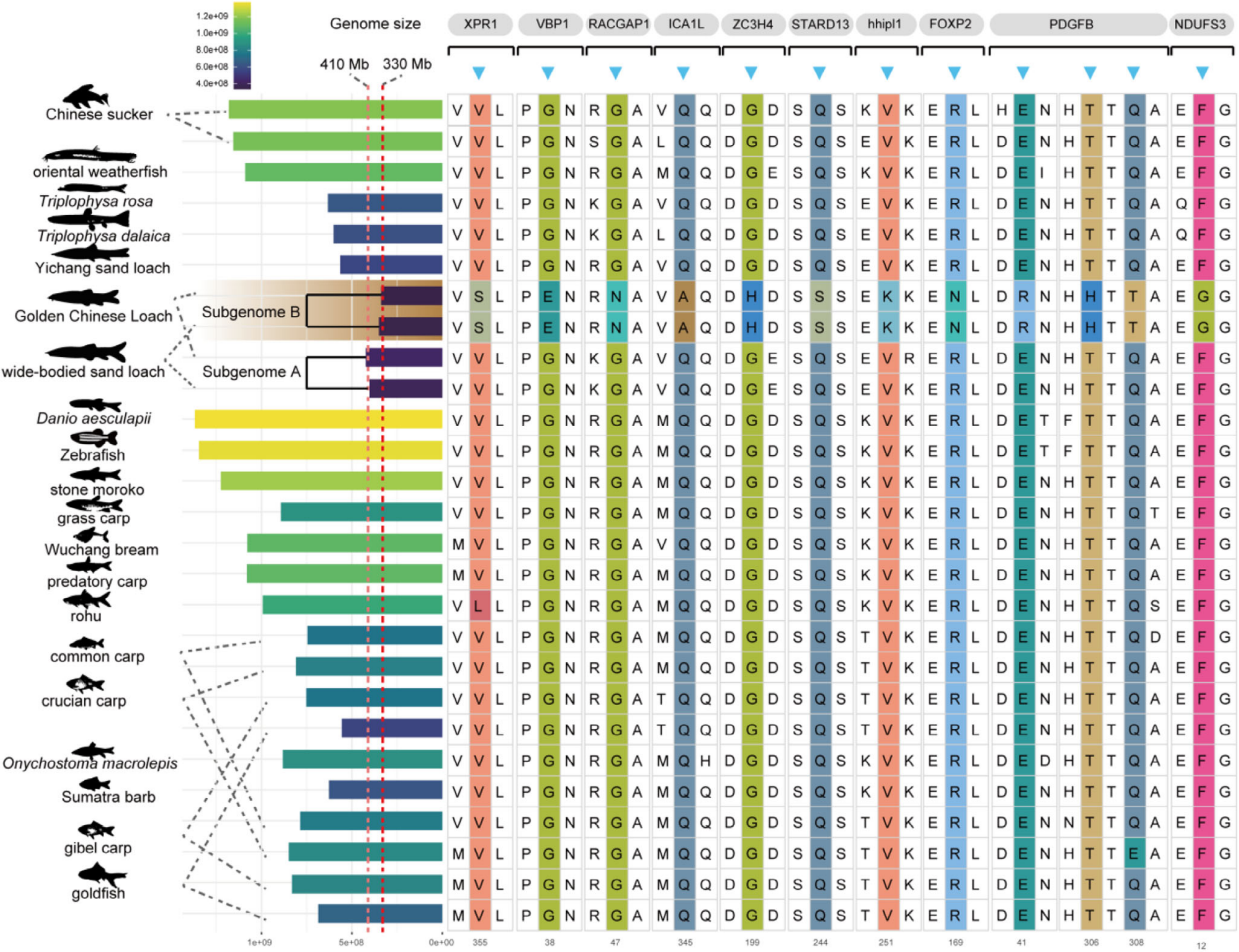
Natural selection in contracted subgenomes of wide‐bodied sand loach and golden Chinese loach. Comparison of haploid genome size and subgenome size among different species in the order Cypriniformes (left). On the right, a list of ten positively selected genes (PSGs) coding for proteins and the selected sites identified in at least two subgenomes from the wide‐bodied sand loach and Golden Chinese Loach.

We obtained four distinct subgenomes after separating the subgenomes of the Golden Chinese Loach and wide‐bodied sand loach. These genomes were relatively small, ranging from 410 to 330 Mb. We grouped these four subgenomes into Group A and Group B (**Figure** **6**). Groups A and B contained one subgenome each from the Golden Chinese Loach and wide‐bodied sand loach, with their relationships determined through evolutionary analysis (Figure 5A).

The size of the group A subgenomes (≈410 Mb) was larger than that of the group B subgenomes (≈330 Mb). The group B subgenomes were the smallest among the genomes in the cypriniform species we studied. To further explore the specific molecular changes associated with this smaller subgenome, we conducted a positive selection analysis target to this subgenome.

A positive selection analysis (see Experimental Section) was used to identify ten genes that showed signs of positive selection. These genes encode the proteins XPR1 (xenotropic and polytropic retrovirus receptor 1), RACGAP1 (Rac GTPase activating protein 1), ICA1L (islet cell autoantigen 1‐like), ZC3H4 (zinc finger CCCH‐type containing 4), STARD13 (StAR‐related lipid transfer domain containing 13), HHIPL1 (hedgehog interacting protein‐like 1), FOXP2 (forkhead box P2), PDGFB (platelet‐derived growth factor beta), and NDUFS3 (NADH: ubiquinone oxidoreductase subunit S3) (Figure 6). These proteins are involved in processes such as cell proliferation, migration, differentiation, metabolic regulation, and signal transduction.^[^ ^45^, ^46^, ^47^, ^48^, ^49^, ^50^, ^51^, ^52^, ^53^, ^54^ ^]^

As the genome size decreases, it is possible that greater genomic instability plays a role, and genes related to genome stability and cell death, such as STARD13 and RACGAP1,^[^ ^55^ ^]^ may play critical roles during the contraction of the genome. Positive selection may have driven the retention or modification of these genes during evolution, helping to maintain proper cell division and reduce genomic instability.

## 3 Conclusion

This study presents chromosome‐level genome assemblies for two loach species, such as Golden Chinese Loach and Yichang Sand Loach, and integrates them with genomic data from 19 previously sequenced cypriniform species, providing significant advancements in understanding the evolutionary dynamics of WGD events within Cypriniformes. The novel subgenome partitioning method (M3) offered a valuable tool for resolving phylogenetic relationships and investigating the molecular mechanisms underlying genomic evolution in polyploid species, particularly in lineages where traditional methods are less effective. These insights advance theoretical frameworks on the evolutionary impacts of polyploidy, enriching our knowledge of genomic evolution and adaptation in vertebrates.

## 4 Experimental Section

### Ethics Statement

All procedures applied in this study were approved by the Institutional Ethics Committee of the Animal Ethical and Welfare Committee of Neijiang Normal University (permit: NJNU20241103), and all methods were carried out following the Code of Practice for the Care and Handling of Animal guidelines. This study complies with the ARRIVE guidelines. All relevant ethical regulations for animal use had been complied with.

### Sample Collection

Samples were collected from artificially bred Yichang Sand Loach and Golden Chinese Loach at the Loach Breeding Base, Neijiang Normal University (Neijiang, China). All samples were adult females, which were confirmed through dissection. Muscle tissue was selected as the source for Nanopore long‐read sequencing, second‐generation sequencing, and construction of the Hi‐C sequencing library.

### Construction of the Sequencing Library

A 20‐kb library was constructed for each species and sequenced on the Oxford Nanopore PromethION platform (Oxford Nanopore Technologies, Oxford, UK) using the standard long‐read sequencing protocol. One PromethION flow cell was used per species, with a theoretical maximum data output of ≈200 Gb per flow cell.

### Short DNA Fragment Library Construction and Sequencing

The libraries for short DNA fragment sequencing were constructed using 1 µg of DNA and the MGIEasy DNA Universal Library Prep Set (MGI Tech Co. Ltd., Shenzhen, China), following the manufacturer's protocol. A unique barcode was added to each sample for identification during sequencing. After constructing the library, the concentration and fragment size distribution of the samples were measured using a Qubit 3.0 Fluorometer (Thermo Fisher Scientific Inc., Pittsburgh, PA, USA) and a Bioanalyzer 2100 system (Agilent Technologies Inc., Santa Clara, CA, USA) to determine an appropriate pooling strategy for sequencing. Libraries passing quality control checks were sequenced on the MGI‐SEQ 2000 platform (MGI Tech Co. Ltd., Shenzhen, China) at the Nanjing Sequencing Center (Novogene Biotechnology Co., Ltd., Nanjing, China).

### Transcriptome Library Construction and Sequencing

Brain, heart, liver, spleen, lung, and kidney tissues were collected from Yichang Sand Loach and Golden Chinese Loach to extract RNA. Total RNA was extracted using the PureLink RNA Mini Kit (Thermo Fisher Scientific Inc.) following the manufacturer's instructions. The quantity, purity, and integrity of the extracted RNA were assessed using a Qubit 3.0 Fluorometer, the NanoDrop Spectrophotometer (Thermo Fisher Scientific Inc.), and an Agilent 2100 Bioanalyzer, respectively. Only samples with a high RNA integrity number (RIN ≥ 8) were selected for sequencing.

RNA fragmentation and PCR amplification were performed to construct the cDNA library according to the RNA‐seq protocol, using a Taq PCR Core Kit (Qiagen GmbH, Hilden, Germany) for amplification. After ensuring all extracted RNA met quality criteria, the RNA from various tissues was combined to maximize the number of expressed genes. RNA sequencing (RNA‐seq) data were generated using the Illumina HiSeq 4000 platform (Illumina Inc., San Diego, CA, USA) with 150 bp paired‐end reads.

### Hi‐C Library Construction and Sequencing

A Hi‐C library was prepared following the manufacturer's protocol and sequenced on the NovaSeq 6000 platform (Illumina Inc.) in paired‐end 150 bp (PE150) mode. Sequencing adapters and low‐quality reads were trimmed to ensure high‐quality data for downstream analyses. All subsequent analyses were performed using the clean data, which was processed to remove low‐quality reads. Raw data were trimmed using Trimmomatic v0.39 software^[^ ^56^ ^]^ following the steps:

- Low‐Quality Read trimming: A sliding window approach was used to trim reads. A window of four bases was evaluated, and reads were trimmed if the average base quality dropped below 15.
- Trimming of Low‐Quality Bases: Reads with quality scores less than 3 at the leading or trailing ends, or containing ‘N’ bases, were trimmed. Parameters used: LEADING: 3, TRAILING: 3.
- Removal of Adapter Contamination: Two modes were used to remove adapter contamination: the simple alignment mode (where the adapter sequence matches with a score of ≥ 10) and the palindrome mode (for read pairs where overlapping base scores > 30).
- Trimming Short Reads: Reads shorter than 36 bases after trimming were discarded.
- Removal of unpaired reads: Unpaired reads that could not form a proper pair were discarded.

### Genome Assembly

*De novo* genome assemblies for the Yichang Sand Loach and Golden Chinese Loach were generated in parallel using the NECAT v0.0.1_update 20200803,^[^ ^57^ ^]^ wtdbg2 v2.5,^[^ ^58^ ^]^ and Flye v2.9‐b1768.^[^ ^59^ ^]^ The best initial assembly, characterized by high contiguity (represented by a high contig N50), was selected. Errors generated during the assembly process were corrected in two rounds using Nanopore reads, followed by Illumina short read polishing with three iterations using NextPolish version 1.4.0.^[^ ^60^ ^]^

Hi‐C reads were aligned to the assembled genome using Chromap v0.2.6,^[^ ^61^ ^]^ and the chromatin interactions between contigs were obtained. Chromosome‐level assembly of the Yichang Sand Loach and Golden Chinese Loach was performed using Yahs v1.1,^[^ ^62^ ^]^ based on the Hi‐C data. To ensure that the chromosomal domains were clearly defined, the Hi‐C reads were mapped back to the chromosome‐level assembly to verify the accuracy of the assembly.

### Repetitive Element Annotation

Genome annotation was initiated by constructing a *de novo* repeat library using RepeatModeler v2.0.1.^[^ ^63^ ^]^ Then, the Repbase database^[^ ^64^ ^]^ was used as a known library to identify repeats specific to the catfish lineages. These libraries were merged and used to search against the assembled genomes using RepeatMasker v4.1.1^[^ ^65^ ^]^ and the parameters “‐nolow ‐gff ‐poly ‐a ‐inv ‐e rmblast” to compile a comprehensive list of repetitive sequences.

### Gene Annotation

Protein‐coding gene regions within the assembled genomes were predicted using the following three approaches:

- *De Novo* prediction: Augustus version 3.3.3 software^[^ ^66^ ^]^ and Snap version 2006‐07‐28 software^[^ ^67^ ^]^ were used to predict the gene models based on the genomic sequence.
- Homolog‐Based prediction: Alignments were performed using TBLASTN v2.11.0^[^ ^68^ ^]^ and Exonerate v2.2.0^[^ ^69^ ^]^ with proteins from seven representative species.
- Transcript‐Based annotation: *de novo* transcriptome assembly was generated using Trinity software package v2.11.0.^[^ ^70^ ^]^ Raw transcripts were aligned to the assembled genome using BLAT v36^[^ ^71^ ^]^ and GMAP v2017‐11‐15,^[^ ^72^ ^]^ followed by validation and integration in the Program to Assemble Spliced Alignments (PASA) pipeline v2.3.3.^[^ ^73^ ^]^

The final gene models were integrated using EvidenceModeler software v1.1.1,^[^ ^74^ ^]^ which combined results from all three approaches and removed erroneous predictions, such as those containing stop codons within coding regions. Functional annotation of the predicted protein‐coding genes was performed using Eggnog‐mapper v2.67,^[^ ^75^ ^]^ providing insight into gene function and the biological processes in which they were involved.

### Gene Family Analysis

To investigate the dynamics of gene family evolution in cyprinid species, protein, and coding gene sequences were collected from several representative cypriniform species and blue catfish as the outgroup species. An all‐*vs*.‐all protein sequence comparisons was performed using BLASTP. Gene families were constructed based on sequence similarity using OrthoMCLight v1.4.^[^ ^76^ ^]^ The number of single‐copy and multi‐copy gene families was determined by examining copy number variations within the clusters.

Single‐copy genes from the identified families were extracted and aligned using the multiple sequence alignment software MAFFT v7.4.75^[^ ^77^ ^]^ to create a super‐length alignment matrix for further phylogenetic analysis. The species tree topology was initially constructed using BI in MrBayes software v3.2.2^[^ ^78^ ^]^ and further confirmed by ML in the Randomized Axelerated Maximum Likelihood (RAxML) tool v8.0.17.^[^ ^79^ ^]^ Both analyses used the GTR + I + G model. The BI analysis consisted of two parallel runs of 2 million generations with three chains each; samples were taken every 100 generations and the first 25% were discarded. ML bootstrapping was performed with 1000 replicates to calculate branch support.

However, concatenation‐based methods can be biased in particular evolutionary scenarios, such as incomplete lineage sorting or gene tree discordance. A coalescent‐based approach was applied to mitigate these potential issues. Individual gene trees were reconstructed using FastTree v2.1.10,^[^ ^80^ ^]^ and then a species tree was inferred using ASTRAL‐IV v1.22.4.6^[^ ^81^ ^]^ by summarizing the topologies of all gene trees. Branch support was evaluated using local posterior probabilities. In cases where the concatenation‐based and coalescent‐based methods yielded conflicting topologies, the coalescent‐based tree was considered more reliable and was retained for downstream analyses.

To estimate divergence times, the species tree was calibrated using MCMCTree software v4.9^[^ ^82^ ^]^ with three time‐calibrated points in the source of TimeTree:^[^ ^83^ ^]^ Node 1: Zebrafish and panther danio (*Danio aesculapii)*, diverging ≈23.0–23.1 million years ago (Mya). Node 2: Catfish and Zebrafish, diverging ≈132.0–170.0 Mya. Node 3: Wuchang bream (*Megalobrama amblycephala*) and predatory carp (*Chanodichthys erythropterus*), diverging ≈0–2.3 Mya.

A log‐normal independent molecular clock model was used to generate 1 000 000 samples and discard the first 25%. Divergence times for each node were visualized using the R package MCMCtreeR v.1.1.^[^ ^84^ ^]^ After splitting the subgenome, annotation information was divided according to the corresponding subgenomes. The same time‐calibration points and analysis pipeline were used to reconstruct the evolutionary relationships of cyprinid species after splitting the subgenome.

### Heterozygous K‐mer Pair Analysis

To investigate the genomic ploidy of the Golden Chinese Loach, the Smudgeplot pipeline^[^ ^85^ ^]^ was utilized to analyze haplotype structures derived from heterozygous k‐mer pairs. Generation of 21‐bp k‐mer frequency data from quality‐trimmed sequencing reads was initiated using KMC v3.0.0.^[^ ^86^ ^]^ Heterozygous k‐mer pairs, differing by a single nucleotide, were systematically identified across the dataset. To minimize the impact of sequencing errors, k‐mers with a depth of less than 20 corresponding to the first local minimum in the k‐mer frequency distribution were excluded. Subsequently, the Smudgeplot R script was applied to visualize the data and infer ploidy levels based on the coverage distribution of heterozygous k‐mer pairs. The pipeline estimates ploidy by comparing the total coverage of each k‐mer pair (CovA + CovB) with the relative contribution of each k‐mer [CovA/(CovA + CovB)].

### Subgenome Partitioning

Subgenome partitioning was performed using MCScanX software^[^ ^87^ ^]^ for a collinearity analysis across all polyploid fish species. Each chromosome was compared to its homologous chromosomes. Based on the homologous relationships, the subgenomes were split using the SubPhaser software v1.2.6,^[^ ^88^ ^]^ leveraging the principle of differential transposon abundance across different subgenomes.

Additionally, the homology between polyploid chromosomes and the chromosomes of closely related diploid species was compared. To determine the *Mash distance* ^[^ ^89^ ^]^ between chromosomes, homologous chromosome pairs between polyploid and diploid species were identified. *Mash* software v2.3^[^ ^89^ ^]^ was used to calculate the Mash distance with the following command: “mash dist ref.fa ‐p 10 ‐s 5000000000 query.fa.” The results were sorted according to the chromosome numbering of the related species and compared with the subgenome splitting results from *SubPhaser* to assess the consistency of the different subgenome partitioning methods.

### Synonymous Substitution Rate (Ks) Analysis

A synteny analysis of the genomes of each species in the order Cypriniformes (with subgenomes treated as independent genomes after splitting) and the blue catfish genome was initially performed using MCScanX software.^[^ ^87^ ^]^ This allowed us to identify syntenic gene pairs within each genome. The protein sequences of the syntenic gene pairs were aligned using the MAFFT program.^[^ ^77^ ^]^ The resulting amino acid alignments were converted into codon‐based nucleotide alignments using the PAL2NAL program.^[^ ^90^ ^]^

The *Ks* (synonymous substitution rate) values for each syntenic gene pair were calculated using KaKs_Calculator v2.0,^[^ ^91^ ^]^ specifically using the Yang–Nielsen method.^[^ ^92^ ^]^ A histogram of *K* values was constructed, with values ranging from 0 to 10 in intervals of 0.1. This histogram was used to compare the distribution of gene pairs across species and identify any significant differences in evolutionary patterns among the species.

### Positively Selected Genes (PSGs)

To identify the PSGs, the CodeML branch‐site model in the PAML software package v4.9^[^ ^82^ ^]^ was used. The null hypothesis constrained the *Ka*/*Ks* value (the non‐synonymous rate to synonymous substitution rate) to 1 at each site on each branch, whereas the alternative hypothesis allowed variable *Ka*/*Ks* values at specific sites within the hibernating clades. The p‐values from the likelihood ratio test (LRT) were calculated to determine whether the alternative hypothesis provided a significantly better fit. PSGs were defined based on a corrected *p*‐value < 0.05 and the presence of at least one positively selected site with a posterior probability > 0.90, as determined by the Bayes empirical Bayes (BEB) analysis.

### Statistical Analysis

All statistical analyses were performed using standard bioinformatics tools and custom scripts. Sequencing data quality was assessed using the Qubit 3.0 Fluorometer and the Agilent Bioanalyzer 2100 to quantify DNA/RNA concentration and integrity. Key metrics, including contig N50, genome completeness (assessed using BUSCO), and chromosome anchoring rates were calculated to evaluate the genome assembly. Repeat content was estimated as a proportion of the genome based on the RepeatMasker output. Phylogenetic support was evaluated using Bayesian posterior probabilities (MrBayes) and maximum likelihood bootstrap values (RAxML), while the reliability of the coalescent‐based species tree was assessed using local posterior probabilities (ASTRAL). Divergence time estimates were reported alongside 95% highest posterior density intervals calculated with MCMCTree. The k‐mer distribution and Smudgeplot profiles were analyzed using default parameters recommended in the Smudgeplot pipeline. LRTs were applied to determine whether the alternative model provided a significantly better fit, with statistical significance assessed via *p*‐values. PSGs were identified based on a false discovery rate‐adjusted *p*‐value < 0.05 and the presence of at least one site under positive selection with a posterior probability > 0.90, as inferred by the BEB analysis.

## Conflict of Interest

The authors declare no conflict of interest.

## Supporting information



Supporting Information

## Data Availability

The genome assembly, mRNA sequences, protein sequences, and gene annotations for *Sinibotia superciliaris* (Golden Chinese Loach) and *Parabotia fasciatus* (Yichang Sand Loach) are available at Figshare at https://doi.org/10.6084/m9.figshare.28520348.

## References

[1] P. Dehal , J. L. Boore , PLoS Biol. 2005, 3, 314. 10.1371/journal.pbio.0030314 PMC1197285 16128622

[2] A. L. Hughes , J. Mol. Evol. 1999, 48, 565. 10198122 10.1007/pl00006499

[3] D. Yu , Y. Ren , M. Uesaka , A. J. S. Beavan , M. Muffato , J. Shen , Y. Li , I. Sato , W. Wan , J. W. Clark , J. N. Keating , E. M. Carlisle , R. P. Dearden , S. Giles , E. Randle , R. S. Sansom , R. Feuda , J. F. Fleming , F. Sugahara , C. Cummins , M. Patricio , W. Akanni , S. D'Aniello , C. Bertolucci , N. Irie , C. Alev , G. Sheng , A. de Mendoza , I. Maeso , M. Irimia , et al.,Nat Ecol Evol 2024, 8, 519. 38216617 10.1038/s41559-023-02299-z PMC10927551

[4] Y. Van de Peer , S. Maere , A. Meyer , Nat. Rev. Genet. 2010, 11, 166. 10.1038/nrg2600-c2 20051987

[5] S. M. Glasauer , S. C. Neuhauss , Mol. Genet. Genomics 2014, 289, 1045. 25092473 10.1007/s00438-014-0889-2

[6] S. Hoegg , H. Brinkmann , J. S. Taylor , A. Meyer , J. Mol. Evol. 2004, 59, 190. 15486693 10.1007/s00239-004-2613-z

[7] V. Ravi , B. Venkatesh , Annu. Rev. Anim. Biosci. 2018, 6, 47. 29447475 10.1146/annurev-animal-030117-014821

[8] C. Canestro , R. Albalat , M. Irimia , J. Garcia‐Fernandez , Semin. Cell Dev. Biol. 2013, 24, 83. 23291262 10.1016/j.semcdb.2012.12.008

[9] Y. Van de Peer , S. Maere , A. Meyer , Nat. Rev. Genet. 2009, 10, 725. 19652647 10.1038/nrg2600

[10] Y. Van de Peer , E. Mizrachi , K. Marchal , Nat. Rev. Genet. 2017, 18, 411. 28502977 10.1038/nrg.2017.26

[11] O. Jaillon , J. M. Aury , F. Brunet , J. L. Petit , N. Stange‐Thomann , E. Mauceli , L. Bouneau , C. Fischer , C. Ozouf‐Costaz , A. Bernot , S. Nicaud , D. Jaffe , S. Fisher , G. Lutfalla , C. Dossat , B. Segurens , C. Dasilva , M. Salanoubat , M. Levy , N. Boudet , S. Castellano , V. Anthouard , C. Jubin , V. Castelli , M. Katinka , B. Vacherie , C. Biemont , Z. Skalli , L. Cattolico , J. Poulain , et al., Nature 2004, 431, 946.15496914

[12] E. Parey , A. Louis , J. Montfort , Y. Guiguen , H. R. Crollius , C. Berthelot , Genome Res. 2022, 32, 1685. 35961774 10.1101/gr.276953.122 PMC9528989

[13] P. Xu , X. Zhang , X. Wang , J. Li , G. Liu , Y. Kuang , J. Xu , X. Zheng , L. Ren , G. Wang , Y. Zhang , L. Huo , Z. Zhao , D. Cao , C. Lu , C. Li , Y. Zhou , Z. Liu , Z. Fan , G. Shan , X. Li , S. Wu , L. Song , G. Hou , Y. Jiang , Z. Jeney , D. Yu , L. Wang , C. Shao , L. Song , et al., Nat. Genet. 2014, 46, 1212. 25240282 10.1038/ng.3098

[14] P. Xu , J. Xu , G. Liu , L. Chen , Z. Zhou , W. Peng , Y. Jiang , Z. Zhao , Z. Jia , Y. Sun , Y. Wu , B. Chen , F. Pu , J. Feng , J. Luo , J. Chai , H. Zhang , H. Wang , C. Dong , W. Jiang , X. Sun , Nat. Commun. 2019, 10, 4625. 31604932 10.1038/s41467-019-12644-1 PMC6789147

[15] J. T. Li , Q. Wang , M. D. Huang Yang , Q. S. Li , M. S. Cui , Z. J. Dong , H. W. Wang , J. H. Yu , Y. J. Zhao , C. R. Yang , Y. X. Wang , X. Q. Sun , Y. Zhang , R. Zhao , Z. Y. Jia , X. Y. Wang , Nat. Genet. 2021, 53, 1493. 34594040 10.1038/s41588-021-00933-9 PMC8492472

[16] D. J. Macqueen , I. A. Johnston , Proc. Biol. Sci. 2014, 281, 20132881. 24452024 10.1098/rspb.2013.2881 PMC3906940

[17] S. Lien , B. F. Koop , S. R. Sandve , J. R. Miller , M. P. Kent , T. Nome , T. R. Hvidsten , J. S. Leong , D. R. Minkley , A. Zimin , F. Grammes , H. Grove , A. Gjuvsland , B. Walenz , R. A. Hermansen , K. von Schalburg , E. B. Rondeau , A. Di Genova , J. K. Samy , J. Olav Vik , M. D. Vigeland , L. Caler , U. Grimholt , S. Jentoft , D. I. Vage , P. de Jong , T. Moen , M. Baranski , Y. Palti , D. R. Smith , et al., Nature 2016, 533, 200. 27088604 10.1038/nature17164 PMC8127823

[18] K. Du , M. Stock , S. Kneitz , C. Klopp , J. M. Woltering , M. C. Adolfi , R. Feron , D. Prokopov , A. Makunin , I. Kichigin , C. Schmidt , P. Fischer , H. Kuhl , S. Wuertz , J. Gessner , W. Kloas , C. Cabau , C. Iampietro , H. Parrinello , C. Tomlinson , L. Journot , J. H. Postlethwait , I. Braasch , V. Trifonov , W. C. Warren , A. Meyer , Y. Guiguen , M. Schartl , Nat Ecol Evol 2020, 4, 841. 32231327 10.1038/s41559-020-1166-x PMC7269910

[19] P. Cheng , Y. Huang , Y. Lv , H. Du , Z. Ruan , C. Li , H. Ye , H. Zhang , J. Wu , C. Wang , R. Ruan , Y. Li , C. Bian , X. You , C. Shi , K. Han , J. Xu , Q. Shi , Q. Wei , Mol. Biol. Evol. 2021, 38, 1595. 33331879 10.1093/molbev/msaa326 PMC8042750

[20] B. Wang , B. Wu , X. Liu , Y. Hu , Y. Ming , M. Bai , J. Liu , K. Xiao , Q. Zeng , J. Yang , H. Wang , B. Guo , C. Tan , Z. Hu , X. Zhao , Y. Li , Z. Yue , J. Mei , W. Jiang , Y. Yang , Z. Li , Y. Gao , L. Chen , J. Jian , H. Du , Genom. Proteom. Bioinform. 2024, 22, qzad002.

[21] L. Yang , G. J. P. Naylor , R. L. Mayden , Mol. Phylogenet. Evol. 2022, 166, 107323. 34634450 10.1016/j.ympev.2021.107323

[22] D. Chen , Q. Zhang , W. Tang , Z. Huang , G. Wang , Y. Wang , J. Shi , H. Xu , L. Lin , Z. Li , W. Chi , L. Huang , J. Xia , X. Zhang , L. Guo , Y. Wang , P. Ma , J. Tang , G. Zhou , M. Liu , F. Liu , X. Hua , B. Wang , Q. Shen , Q. Jiang , J. Lin , X. Chen , H. Wang , M. Dou , L. Liu , et al., Proc. Natl. Acad. Sci. USA 2020, 117, 29775. 33139555 10.1073/pnas.2005545117 PMC7703540

[23] Z. Chen , Y. Omori , S. Koren , T. Shirokiya , T. Kuroda , A. Miyamoto , H. Wada , A. Fujiyama , A. Toyoda , S. Zhang , T. G. Wolfsberg , K. Kawakami , A. M. Phillippy , N. C. S. Program , J. C. Mullikin , S. M. Burgess , Sci. Adv. 2019, 5, aav0547. 10.1126/sciadv.aav0547 PMC6594761 31249862

[24] M. R. Xu , Z. Y. Liao , J. R. Brock , K. Du , G. Y. Li , Z. Q. Chen , Y. H. Wang , Z. N. Gao , G. Agarwal , K. H. Wei , F. Shao , S. Pang , A. E. Platts , J. van de Velde , H. M. Lin , S. J. Teresi , K. Bird , C. E. Niederhuth , J. G. Xu , G. H. Yu , J. Y. Yang , S. F. Dai , A. Nelson , I. Braasch , X. G. Zhang , M. Schartl , P. P. Edger , M. J. Han , H. H. Zhang , Nat. Commun. 2023, 14, 8357. 38102128 10.1038/s41467-023-43740-y PMC10724154

[25] L. Yang , T. Sado , M. Vincent Hirt , E. Pasco‐Viel , M. Arunachalam , J. Li , X. Wang , J. Freyhof , K. Saitoh , A. M. Simons , M. Miya , S. He , R. L. Mayden , Mol. Phylogenet. Evol. 2015, 85, 97. 25698355 10.1016/j.ympev.2015.01.014

[26] V. Bitsikas , F. Cubizolles , A. F. Schier , Curr. Biol. 2024, 34, 1532. 38490200 10.1016/j.cub.2024.02.022

[27] L. Yang , R. L. Mayden , G. J. P. Naylor , Biology 2025, 14, 531. 40427720 10.3390/biology14050531 PMC12109351

[28] C. S. Tsigenopoulos , P. Rab , D. Naran , P. Berrebi , Heredity 2002, 88, 466. 12180089 10.1038/sj.hdy.6800080

[29] Y. Lv , Y. Li , Y. Huang , J. Wang , Z. Tian , Y. He , J. Shi , Z. Huang , Z. Wen , Q. J. A. R. Shi , Aquac. Rep. 2024, 35, 102033.

[30] X. X. Shen , J. L. Steenwyk , A. Rokas , Syst. Biol. 2021, 70, 997. 33616672 10.1093/sysbio/syab011

[31] G. Reynolds , B. Mumey , V. Strnadova‐Neeley , J. Lachowiec , Appl. Plant Sci. 2024, 12, 11581. 10.1002/aps3.11581 PMC11342227 39184200

[32] H. Kuhl , K. Du , M. Schartl , L. Kalous , M. Stock , D. K. Lamatsch , Nat. Commun. 2022, 13, 4092. 35835759 10.1038/s41467-022-31515-w PMC9283417

[33] H. Fan , A. R. Ives , Y. Surget‐Groba , C. H. Cannon , BMC Genomics 2015, 16, 522. 26169061 10.1186/s12864-015-1647-5 PMC4501066

[34] A. M. Session , Trends Genet. 2024, 40, 621. 38637269 10.1016/j.tig.2024.03.008

[35] V. Slechtova , J. Bohlen , J. Freyhof , P. Rab , Mol. Phylogenet. Evol. 2006, 39, 529. 16337410 10.1016/j.ympev.2005.09.018

[36] G. Tu , Z. Yan , L. Zhang , Z. Liu , Y. Lv , Z. Li , J. He , S. Weng , J. He , M. Wang , Sci Data 2025, 12, 285. 39962122 10.1038/s41597-025-04615-7 PMC11832761

[37] Y. Wang , X. Y. Li , W. J. Xu , K. Wang , B. Wu , M. Xu , Y. Chen , L. J. Miao , Z. W. Wang , Z. Li , X. J. Zhang , Z. Yin , B. T. Zhou , Y. L. Yang , C. L. Zhu , M. L. Hu , J. M. Zheng , C. G. Feng , Q. Qiu , L. T. Tian , M. Lu , F. Peng , W. J. Lu , J. F. Tong , J. G. Tong , B. D. Fu , P. Yu , M. Ding , R. H. Gan , Q. Q. Zhang , et al., Nat. Ecol. Evol. 2022, 6, 1354. 35817827 10.1038/s41559-022-01813-z PMC9439954

[38] J. F. Gui , Water Biol. Secur. 2024, 3, 100271.

[39] T. Wang , Y. P. Zhang , Z. Y. Yang , Z. Liu , Y. Y. Du , BMC Evol. Biol. 2020, 20, 151. 33183225 10.1186/s12862-020-01718-0 PMC7663858

[40] L. Ding , J. Liao , N. Liu , Ecol. Evol. 2020, 10, 1722. 32076546 10.1002/ece3.6008 PMC7029067

[41] M. Xia , Y. Liu , J. Liu , D. Chen , Y. Shi , Z. Chen , D. Chen , R. Jin , H. Chen , S. Zhu , P. Li , J. Si , Y. Qiu , Mol. Phylogenet. Evol. 2022, 169, 107431. 35131418 10.1016/j.ympev.2022.107431

[42] Z. B. Wang , T. N. Oo , L. P. Zheng , X. Y. Chen , Ecol. Evol. 2024, 14, 70448. 10.1002/ece3.70448 PMC11569833 39554884

[43] W. Chi , X. Ma , J. Niu , M. Zou , BMC Genomics 2017, 18, 310. 28427344 10.1186/s12864-017-3703-9 PMC5397779

[44] S. Liu , J. Luo , J. Chai , L. Ren , Y. Zhou , F. Huang , X. Liu , Y. Chen , C. Zhang , M. Tao , B. Lu , W. Zhou , G. Lin , C. Mai , S. Yuan , J. Wang , T. Li , Q. Qin , H. Feng , K. Luo , J. Xiao , H. Zhong , R. Zhao , W. Duan , Z. Song , Y. Wang , J. Wang , L. Zhong , L. Wang , Z. Ding , et al., Proc. Natl. Acad. Sci. USA 2016, 113, 1327. 26768847 10.1073/pnas.1512955113 PMC4747765

[45] D. Aravani , G. E. Morris , P. D. Jones , H. K. Tattersall , E. Karamanavi , M. A. Kaiser , R. B. Kostogrys , M. Ghaderi Najafabadi , S. L. Andrews , M. Nath , S. Ye , E. J. Stringer , N. J. Samani , T. R. Webb , Circulation 2019, 140, 500. 31163988 10.1161/CIRCULATIONAHA.119.041059 PMC6686954

[46] D. P. Bondeson , B. R. Paolella , A. Asfaw , M. V. Rothberg , T. A. Skipper , C. Langan , G. Mesa , A. Gonzalez , L. E. Surface , K. Ito , M. Kazachkova , W. N. Colgan , A. Warren , J. M. Dempster , J. M. Krill‐Burger , M. Ericsson , A. A. Tang , I. Fung , E. S. Chambers , M. Abdusamad , N. Dumont , J. G. Doench , F. Piccioni , D. E. Root , J. Boehm , W. C. Hahn , M. Mannstadt , J. M. McFarland , F. Vazquez , T. R. Golub , Nat. Cancer 2022, 3, 681. 35437317 10.1038/s43018-022-00360-7 PMC9246846

[47] C. Estell , L. Davidson , P. C. Steketee , A. Monier , S. West , Elife 2021, 10, 67305. 10.7554/eLife.67305 PMC8137146 33913806

[48] J. He , M. Xia , W. H. Tsang , K. L. Chow , J. Xia , J. Cell Sci. 2015, 128, 3822. 26306493 10.1242/jcs.173534

[49] H. Miao , W. Gao , L. Zhong , H. Li , D. Chen , C. Xu , Z. Wen , Y. Chen , Hum. Cell 2024, 37, 1141. 38700744 10.1007/s13577-024-01068-9 PMC11194215

[50] J. M. Piek , Int. J. Gynecol. Cancer 2013, 23, 1348. 10.1097/01.IGC.0000434437.52819.d2 24257547

[51] P. Wang , X. Cheng , Z. Fu , C. Zhou , W. Lu , X. Xie , Int. J. Gynecol. Cancer 2013, 23, 622. 23446378 10.1097/IGC.0b013e318287a90d

[52] B. D. Wilson , L. J. Ricks‐Santi , T. E. Mason , M. Abbas , R. A. Kittles , G. M. Dunston , Y. M. Kanaan , Cancer Genomics Proteom 2018, 15, 185. 10.21873/cgp.20076 PMC5971010 29695400

[53] R. Wu , S. Gandhi , Y. Tokumaru , M. Asaoka , M. Oshi , L. Yan , T. Ishikawa , K. Takabe , Breast Cancer Res. Treat. 2022, 195, 17. 35793004 10.1007/s10549-022-06661-w PMC12118772

[54] X. Yan , H. Zhou , T. Zhang , P. Xu , S. Zhang , W. Huang , L. Yang , X. Gu , R. Ni , T. Zhang , Tumour Biol. 2015, 36, 9611. 26142732 10.1007/s13277-015-3701-y

[55] H. He , J. Huang , S. Wu , S. Jiang , L. Liang , Y. Liu , W. Liu , L. Xie , Y. Tao , Y. Jiang , L. Cong , J. Hematol. Oncol. 2021, 14, 171. 34663417 10.1186/s13045-021-01184-1 PMC8524929

[56] A. M. Bolger , M. Lohse , B. Usadel , Bioinformatics 2014, 30, 2114. 24695404 10.1093/bioinformatics/btu170 PMC4103590

[57] Y. Chen , F. Nie , S. Q. Xie , Y. F. Zheng , Q. Dai , T. Bray , Y. X. Wang , J. F. Xing , Z. J. Huang , D. P. Wang , L. J. He , F. Luo , J. X. Wang , Y. Z. Liu , C. L. Xiao , Nat. Commun. 2021, 12, 60. 33397900 10.1038/s41467-020-20236-7 PMC7782737

[58] J. Ruan , H. Li , Nat. Methods 2020, 17, 155. 31819265 10.1038/s41592-019-0669-3 PMC7004874

[59] M. Kolmogorov , J. Yuan , Y. Lin , P. A. Pevzner , Nat. Biotechnol. 2019, 37, 540. 30936562 10.1038/s41587-019-0072-8

[60] J. Hu , J. Fan , Z. Sun , S. Liu , Bioinformatics 2020, 36, 2253. 31778144 10.1093/bioinformatics/btz891

[61] H. Zhang , L. Song , X. Wang , H. Cheng , C. Wang , C. A. Meyer , T. Liu , M. Tang , S. Aluru , F. Yue , X. S. Liu , H. Li , Nat. Commun. 2021, 12, 6566. 34772935 10.1038/s41467-021-26865-w PMC8589834

[62] C. Zhou , S. A. McCarthy , R. Durbin , Bioinformatics 2023, 39, btac808. 36525368 10.1093/bioinformatics/btac808 PMC9848053

[63] J. M. Flynn , R. Hubley , C. Goubert , J. Rosen , A. G. Clark , C. Feschotte , A. F. Smit , Proc. Natl. Acad. Sci. USA 2020, 117, 9451. 32300014 10.1073/pnas.1921046117 PMC7196820

[64] J. Jurka , V. V. Kapitonov , A. Pavlicek , P. Klonowski , O. Kohany , J. Walichiewicz , Cytogenet. Genome Res. 2005, 110, 462. 16093699 10.1159/000084979

[65] M. Tarailo‐Graovac , N. Chen , Curr. Protoc. Bioinformatics 2009, 4, 4.10.11. 10.1002/0471250953.bi0410s25 19274634

[66] M. Stanke , O. Keller , I. Gunduz , A. Hayes , S. Waack , B. Morgenstern , Nucleic Acids Res. 2006, 34, W435. 16845043 10.1093/nar/gkl200 PMC1538822

[67] I. Korf , BMC Bioinformatics 2004, 5, 59. 15144565 10.1186/1471-2105-5-59 PMC421630

[68] E. M. Gertz , Y. K. Yu , R. Agarwala , A. A. Schaffer , S. F. Altschul , BMC Biol. 2006, 4, 41. 17156431 10.1186/1741-7007-4-41 PMC1779365

[69] G. S. Slater , E. Birney , BMC Bioinformatics 2005, 6, 31. 15713233 10.1186/1471-2105-6-31 PMC553969

[70] M. G. Grabherr , B. J. Haas , M. Yassour , J. Z. Levin , D. A. Thompson , I. Amit , X. Acidness , L. Fan , R. Raychowdhury , Q. Zeng , Z. Chen , E. Mauceli , N. Hacohen , A. Gnirke , N. Rhind , F. di Palma , B. W. Birren , C. Nusbaum , K. Lindblad‐Toh , N. Friedman , A. Regev , Nat. Biotechnol. 2011, 29, 644. 21572440 10.1038/nbt.1883 PMC3571712

[71] W. J. Kent , Genome Res. 2002, 12, 656. 11932250 10.1101/gr.229202 PMC187518

[72] T. D. Wu , C. K. Watanabe , Bioinformatics 2005, 21, 1859. 15728110 10.1093/bioinformatics/bti310

[73] B. J. Haas , A. L. Delcher , S. M. Mount , J. R. Wortman , R. K. Smith Jr. , L. I. Hannick , R. Maiti , C. M. Ronning , D. B. Rusch , C. D. Town , S. L. Salzberg , O. White , Nucleic Acids Res. 2003, 31, 5654. 14500829 10.1093/nar/gkg770 PMC206470

[74] B. J. Haas , S. L. Salzberg , W. Zhu , M. Pertea , J. E. Allen , J. Orvis , O. White , C. R. Buell , J. R. Wortman , Genome Biol. 2008, 9, R7. 18190707 10.1186/gb-2008-9-1-r7 PMC2395244

[75] C. P. Cantalapiedra , A. Hernandez‐Plaza , I. Letunic , P. Bork , J. Huerta‐Cepas , Mol. Biol. Evol. 2021, 38, 5825. 34597405 10.1093/molbev/msab293 PMC8662613

[76] L. Li , C. J. Stoeckert Jr. , D. S. Roos , Genome Res. 2003, 13, 2178. 12952885 10.1101/gr.1224503 PMC403725

[77] K. Katoh , D. M. Standley , Mol. Biol. Evol. 2013, 30, 772. 23329690 10.1093/molbev/mst010 PMC3603318

[78] F. Ronquist , M. Teslenko , P. van der Mark , D. L. Ayres , A. Darling , S. Hohna , B. Larget , L. Liu , M. A. Sutured , J. P. Huelsenbeck , Syst. Biol. 2012, 61, 539. 22357727 10.1093/sysbio/sys029 PMC3329765

[79] A. Stamatakis , Bioinformatics 2014, 30, 1312. 24451623 10.1093/bioinformatics/btu033 PMC3998144

[80] M. N. Price , P. S. Dehal , A. P. Arkin , PLoS One 2010, 5, 9490. 10.1371/journal.pone.0009490 PMC2835736 20224823

[81] C. Zhang , S. Mirarab , Mol. Biol. Evol. 2022, 39, msac215. 36201617 10.1093/molbev/msac215 PMC9750496

[82] Z. Yang , Mol. Biol. Evol. 2007, 24, 1586. 17483113 10.1093/molbev/msm088

[83] S. Kumar , G. Stecher , M. Suleski , S. B. Hedges , Mol. Biol. Evol. 2017, 34, 1812. 28387841 10.1093/molbev/msx116

[84] M. N. Puttick , Bioinformatics 2019, 35, 5321. 31292621 10.1093/bioinformatics/btz554

[85] T. R. Ranallo‐Benavidez , K. S. Jaron , M. C. Schatz , Nat. Commun. 2020, 11, 1432. 32188846 10.1038/s41467-020-14998-3 PMC7080791

[86] M. Kokot , M. Dlugosz , S. Deorowicz , Bioinformatics 2017, 33, 2759. 28472236 10.1093/bioinformatics/btx304

[87] Y. Wang , H. Tang , J. D. Debarry , X. Tan , J. Li , X. Wang , T. H. Lee , H. Jin , B. Marler , H. Guo , J. C. Kissinger , A. H. Paterson , Nucleic Acids Res. 2012, 40, 49. 10.1093/nar/gkr1293 PMC3326336 22217600

[88] K. H. Jia , Z. X. Wang , L. Wang , G. Y. Li , W. Zhang , X. L. Wang , F. J. Xu , S. Q. Jiao , S. S. Zhou , H. Liu , Y. Ma , G. Bi , W. Zhao , Y. A. El‐Kassaby , I. Porth , G. Li , R. G. Zhang , J. F. Mao , New Phytol. 2022, 235, 801. 35460274 10.1111/nph.18173

[89] B. D. Ondov , T. J. Treangen , P. Melsted , A. B. Mallonee , N. H. Bergman , S. Koren , A. M. Phillippy , Genome Biol. 2016, 17, 132. 27323842 10.1186/s13059-016-0997-x PMC4915045

[90] M. Suyama , D. Torrents , P. Bork , Nucleic Acids Res. 2006, 34, W609. 16845082 10.1093/nar/gkl315 PMC1538804

[91] D. Wang , Y. Zhang , Z. Zhang , J. Zhu , J. Yu , Genom. Proteom. Bioinform. 2010, 8, 77. 10.1016/S1672-0229(10)60008-3 PMC5054116 20451164

[92] Z. Yang , R. Nielsen , Mol. Biol. Evol. 2000, 17, 32. 10666704 10.1093/oxfordjournals.molbev.a026236

