# Supplementary material for: Subgenome Partitioning and Polyploid Genome Evolution in the Loach Family Botiidae (Order Cypriniformes)
Source: Adv Sci (Weinh). 2025 Jul 7;12(36):e05411. doi: 10.1002/advs.202505411 (PMC12462953; doi:10.1002/advs.202505411)
Supplement: Supplementary file 1 — Supporting Information [file ADVS-12-e05411-s001.docx]

Supporting Information

Subgenome Partitioning and Polyploid Genome Evolution in the Loach Family Botiidae (Order Cypriniformes)

Yunyun Lv*, Jia Li, Yanping Li, Yu Huang, Qiang Lai, Zhengyong Wen, Jun Wang, Yang He, Jinrong Shi，Zejin Huang, Ying Jiang, Yves Van de Peer*, Qiong Shi*, Biwen Xie*, Yongming Wang*


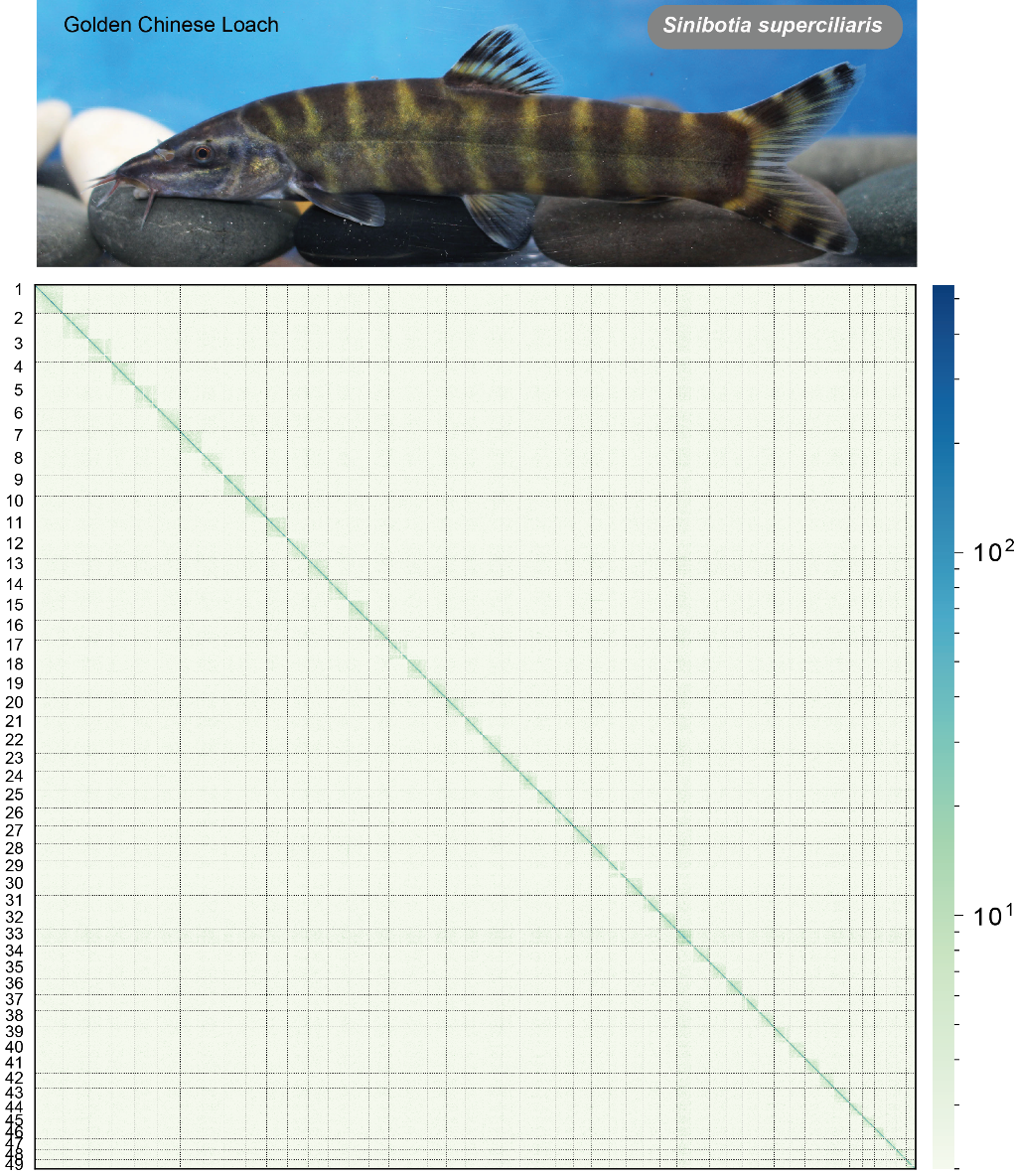


Figure S1. Image of the Golden Chinese Loach (top) and the degree of interaction between different chromosomes (bottom).


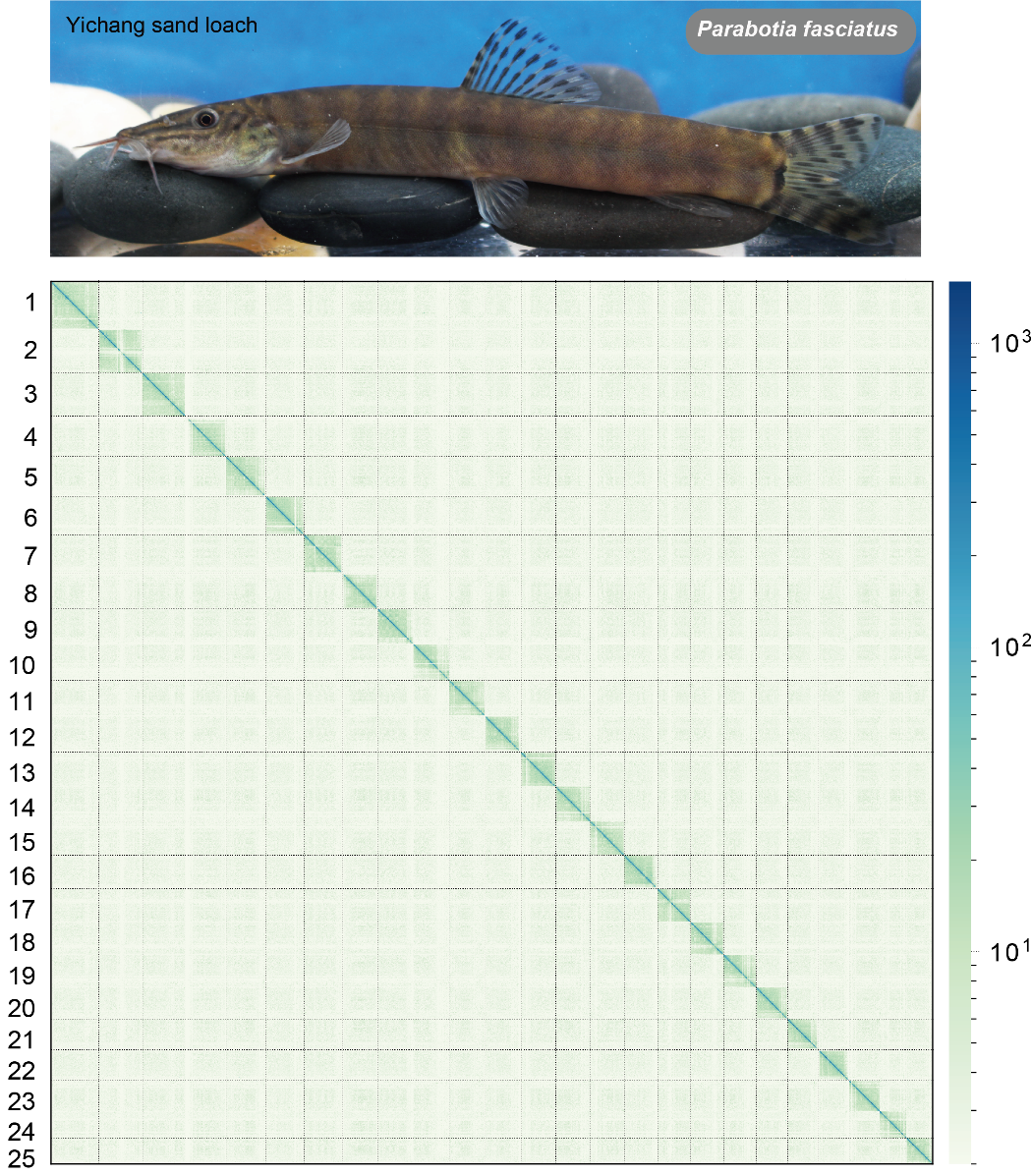


Figure S2. Image of the Yichang Sand Loach (top) and the degree of interaction between different chromosomes (bottom).


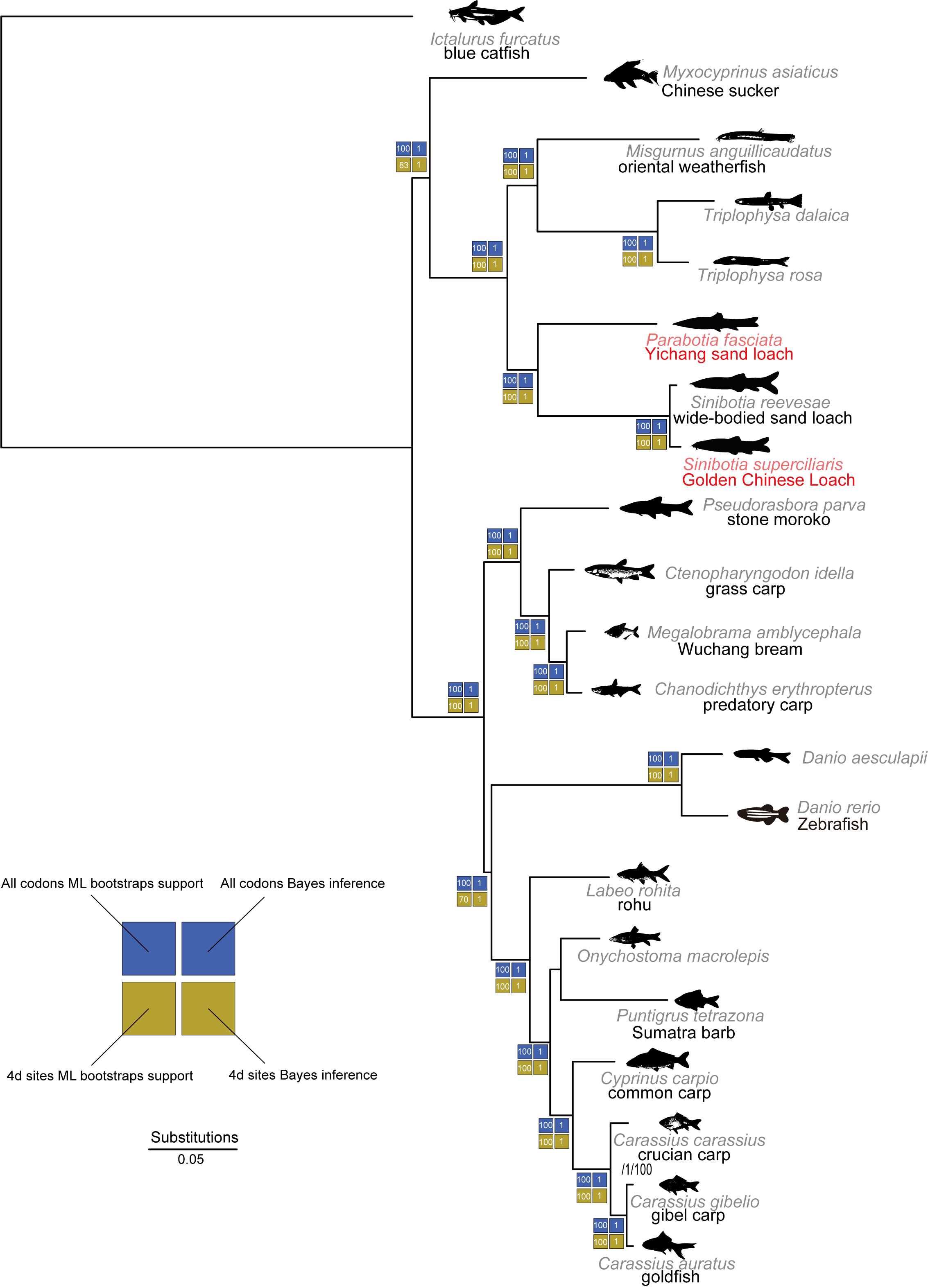


Figure S3. Image of the Yichang Sand Loach (top) and the degree of interaction between different chromosomes (bottom).


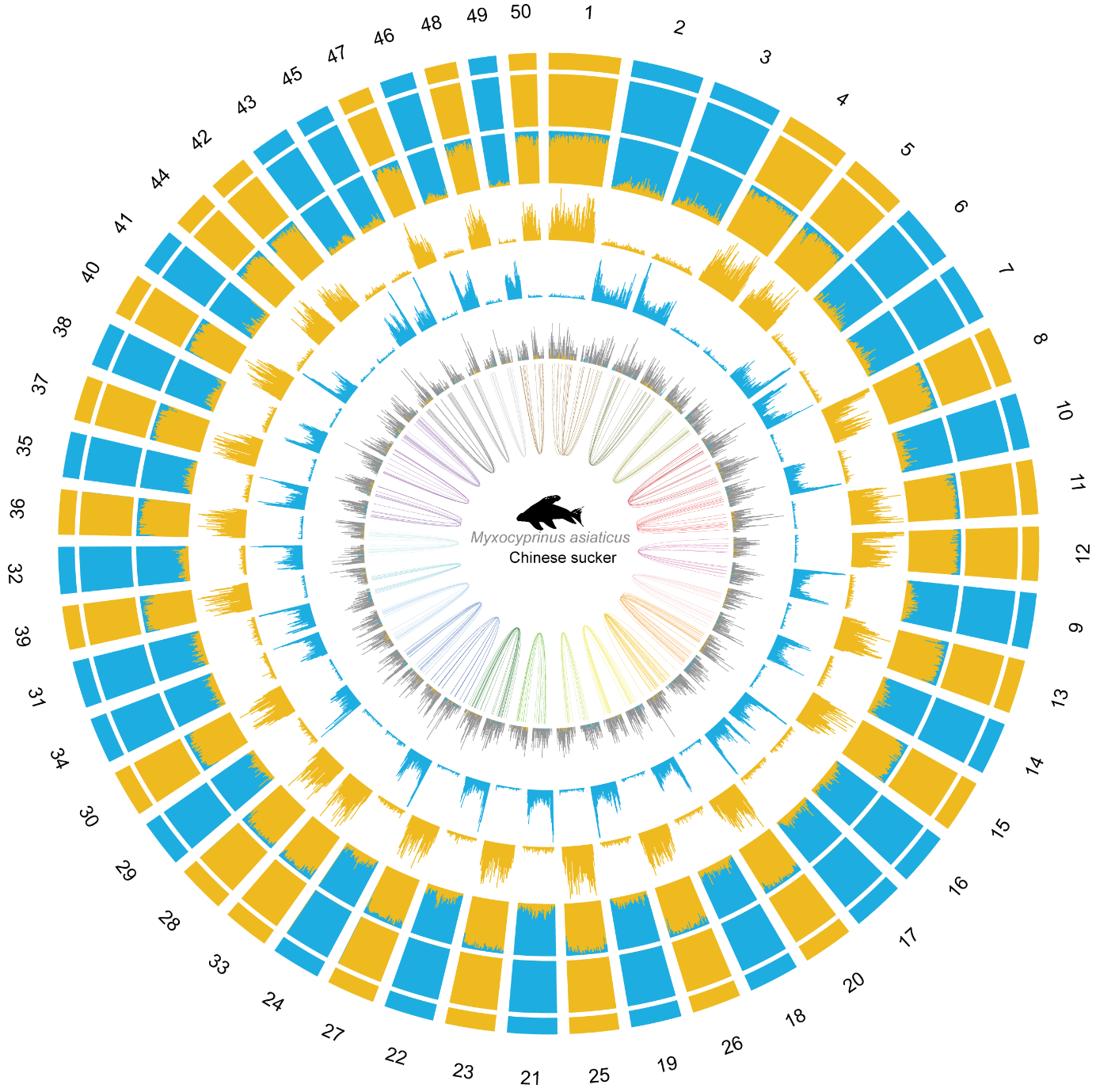


Figure S4. The subgenomes of the Chinese sucker species were successfully identified using the SubPhaser software. The two subgenomes are visualized in distinct colors: yellow and blue.


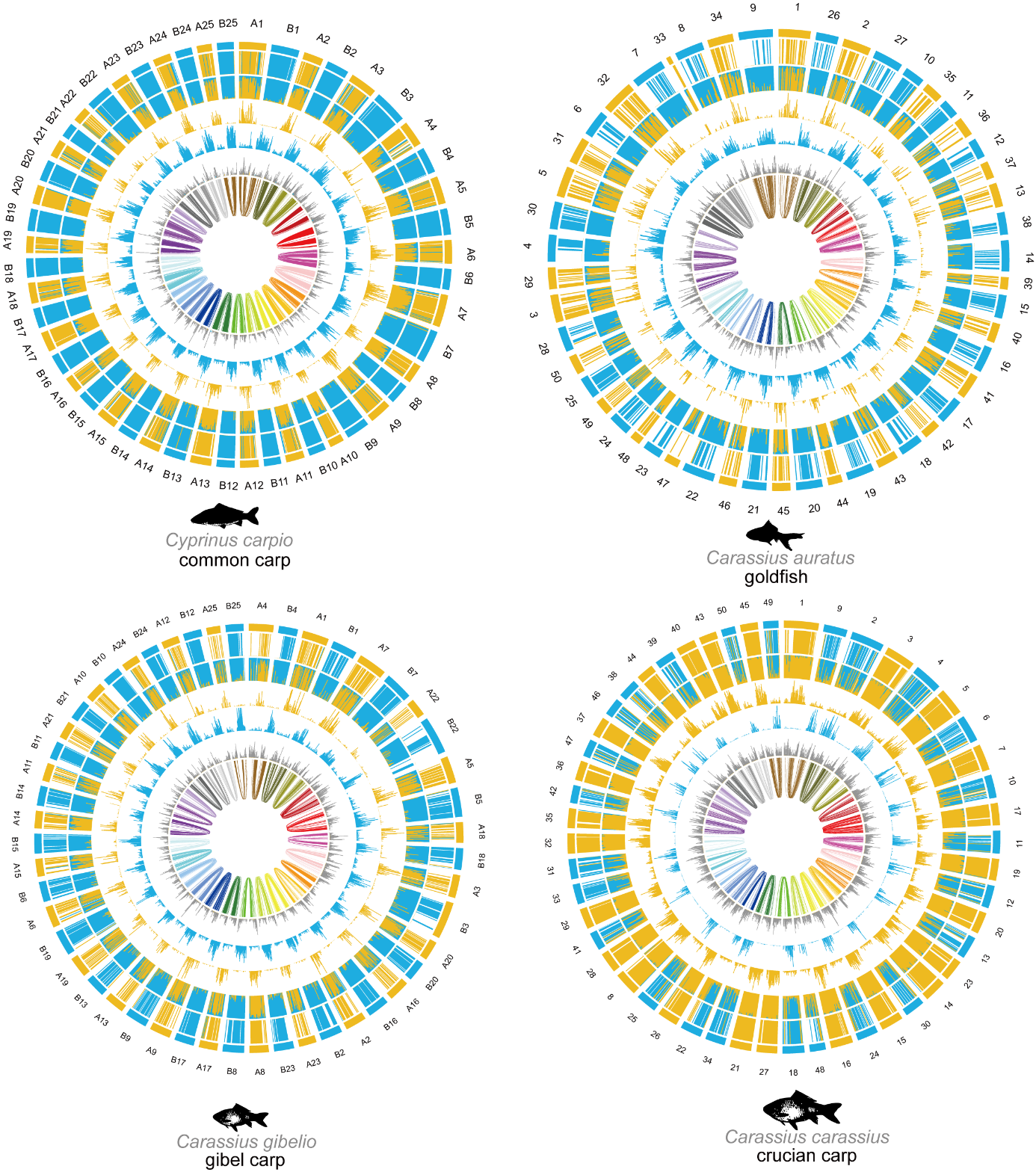


Figure S5. The subgenomes of the carp species were successfully identified using the SubPhaser software and are displayed in yellow and blue.


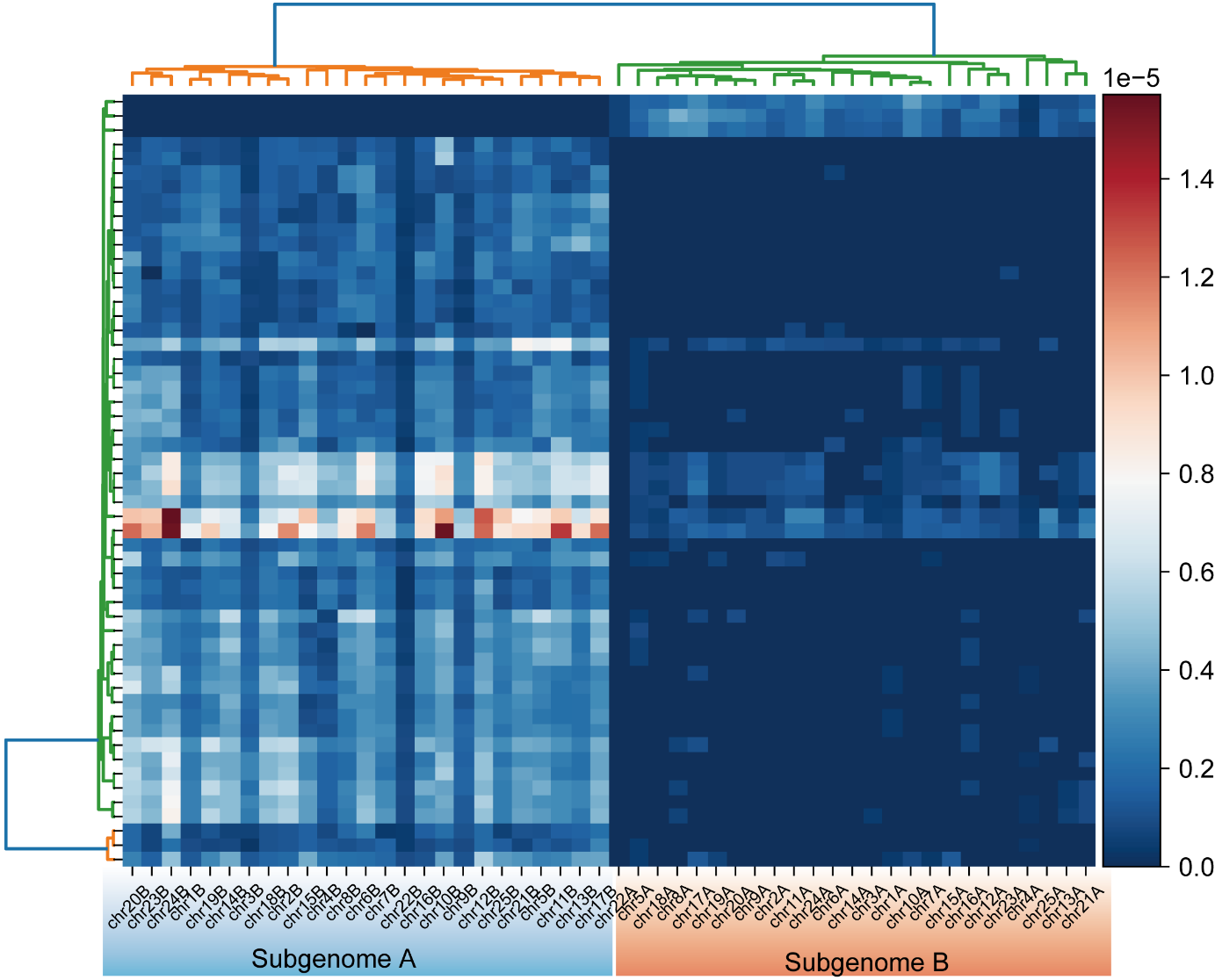


Figure S6. Heatmap of Subgenome Partitioning in the Goldfish Genome (ASM1972071v2) Identified Using Subphraser. The heatmap displays the correlation of the repeat-type k-mer occurrence frequencies across different chromosomes, with color intensity indicating the degree of similarity.


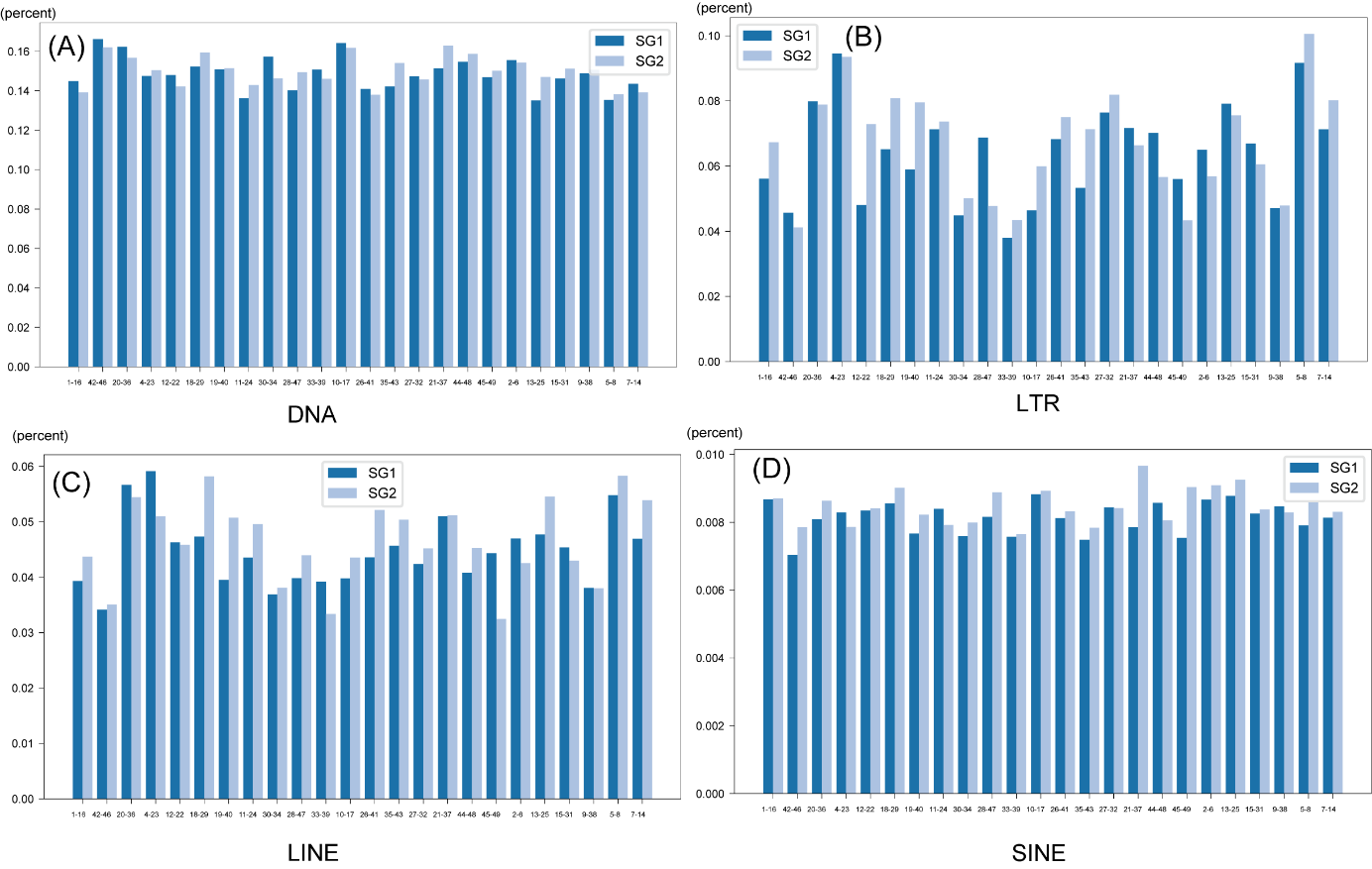


Figure S7. Comparison of Transposon Content among Homologous Chromosomes in the Wide-bodied Sand Loach. (A) DNA Transposons; (B) LTR Retrotransposons; (C) LINEs; (D) SINEs


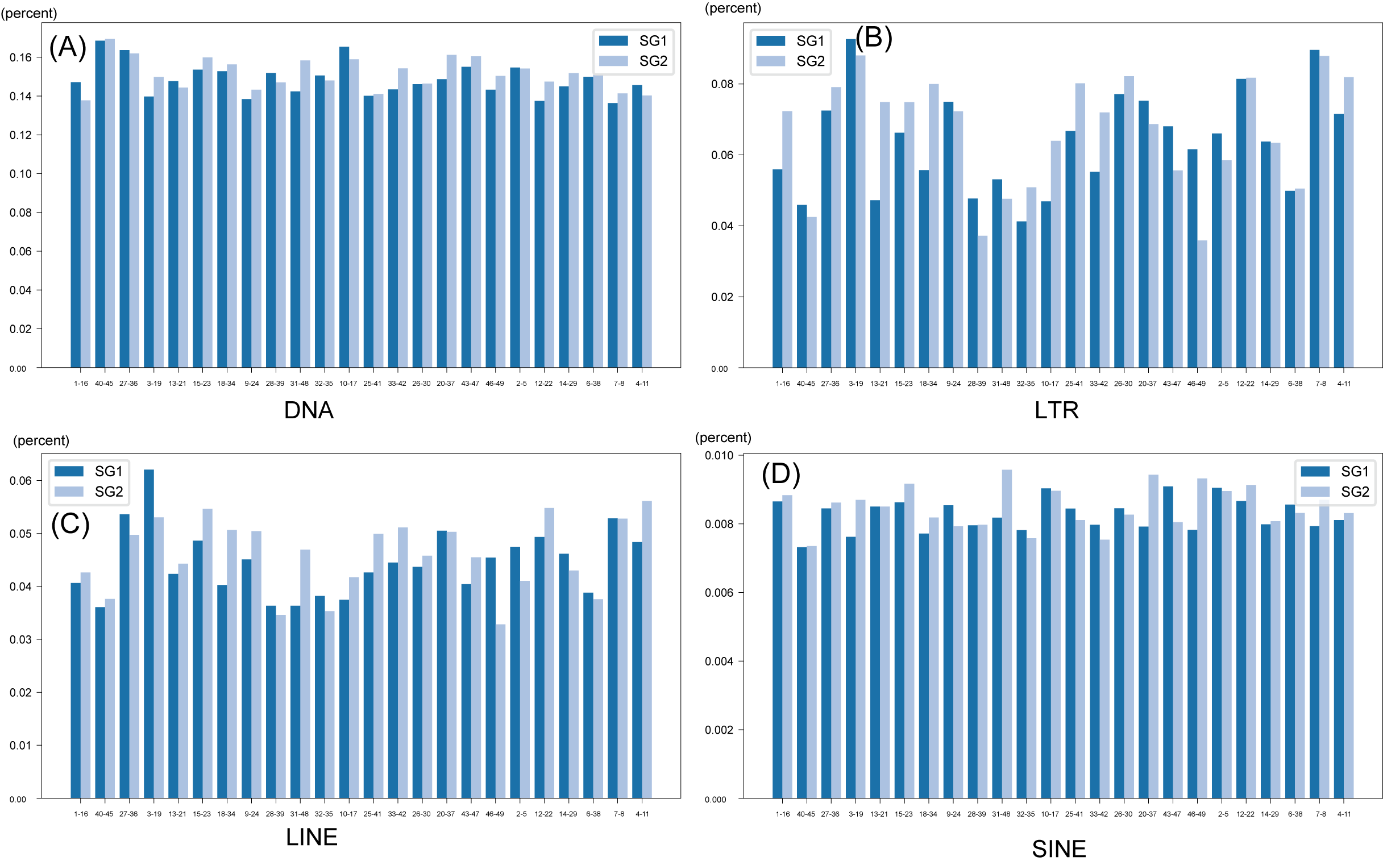


Figure S8. Comparison of Transposon Content among Homologous Chromosomes in the Golden Chinese Loach. (A) DNA Transposons; (B) LTR Retrotransposons; (C) LINEs; (D) SINEs


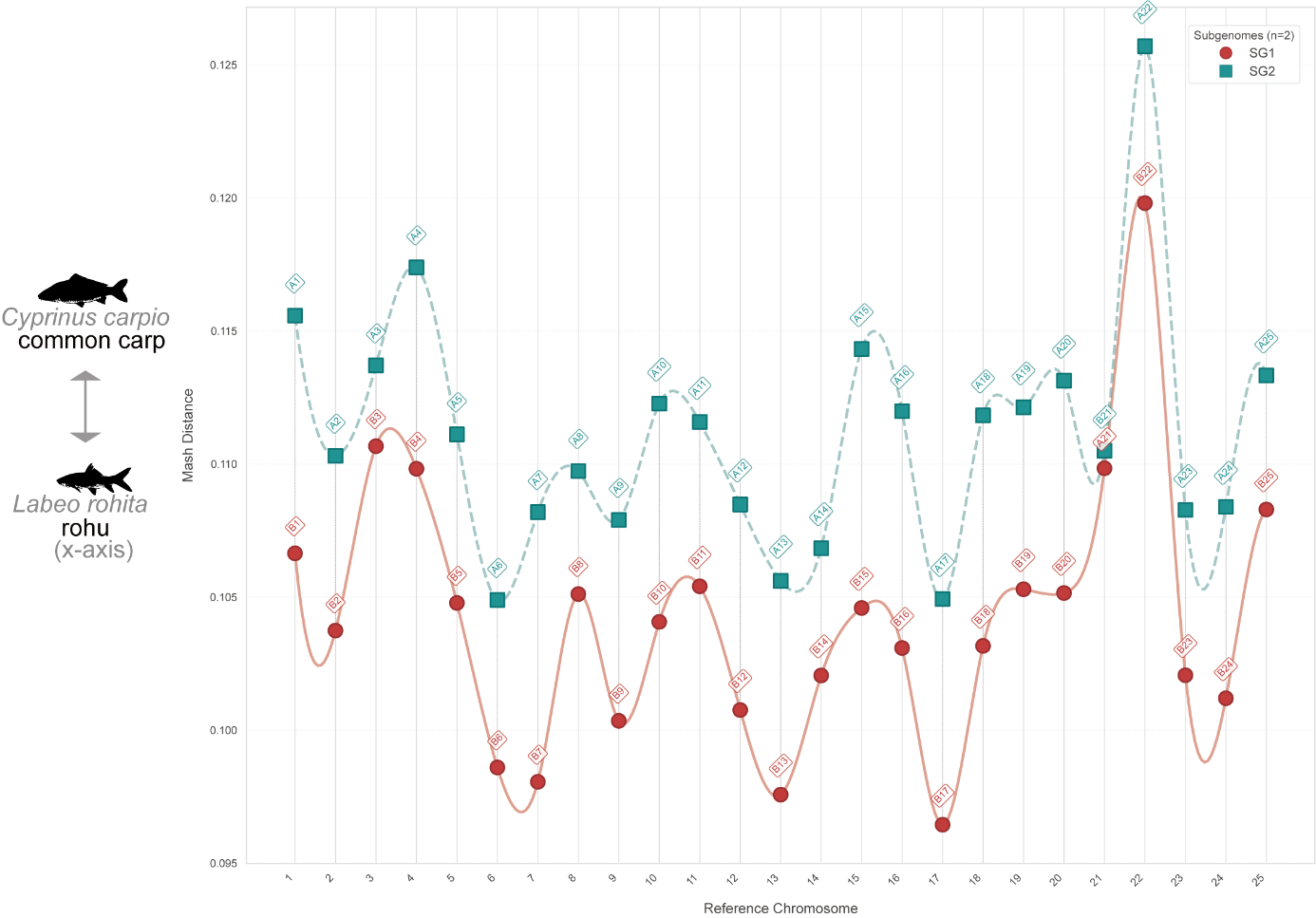


Figure S9. Subgenome Partitioning of the Common Carp Identified Using the M3 Method with Rohu as the Reference Genome


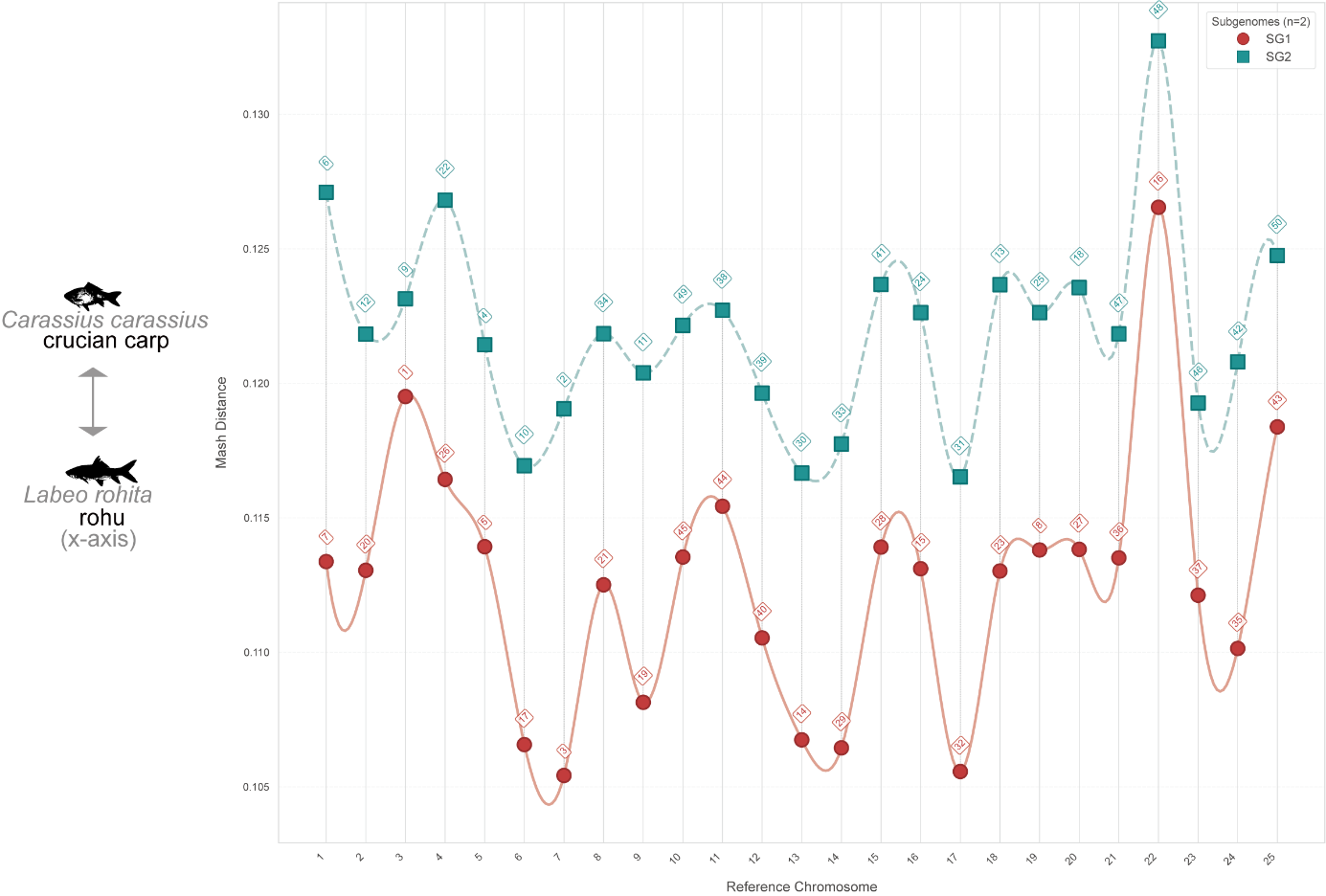


Figure S10. Subgenome Partitioning of the Crucian Carp Identified Using the M3 Method with Rohu as the Reference Genome


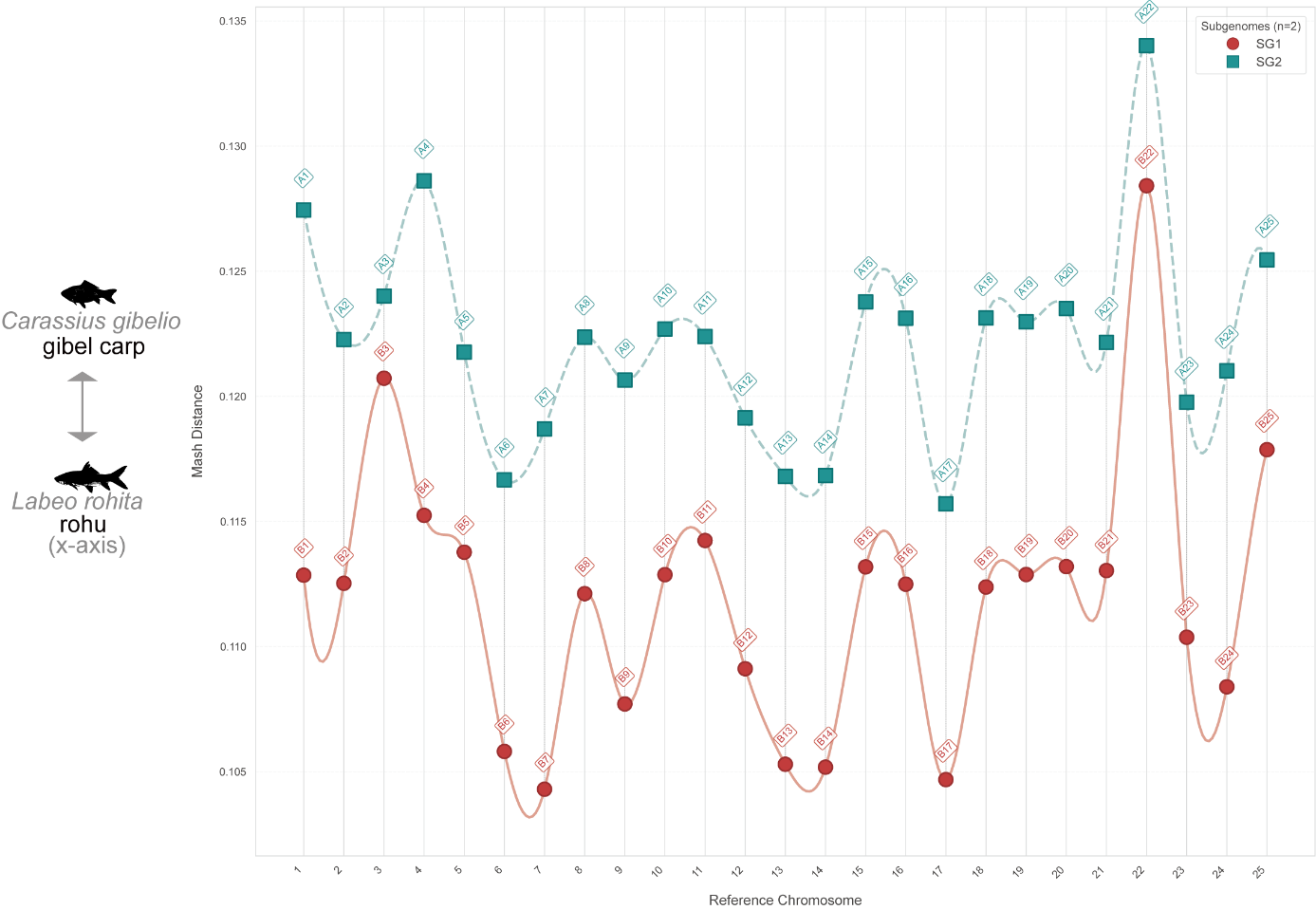


Figure S11. Subgenome Partitioning of Gibel Carp Identified Using the M3 Method with Rohu as the Reference Genome


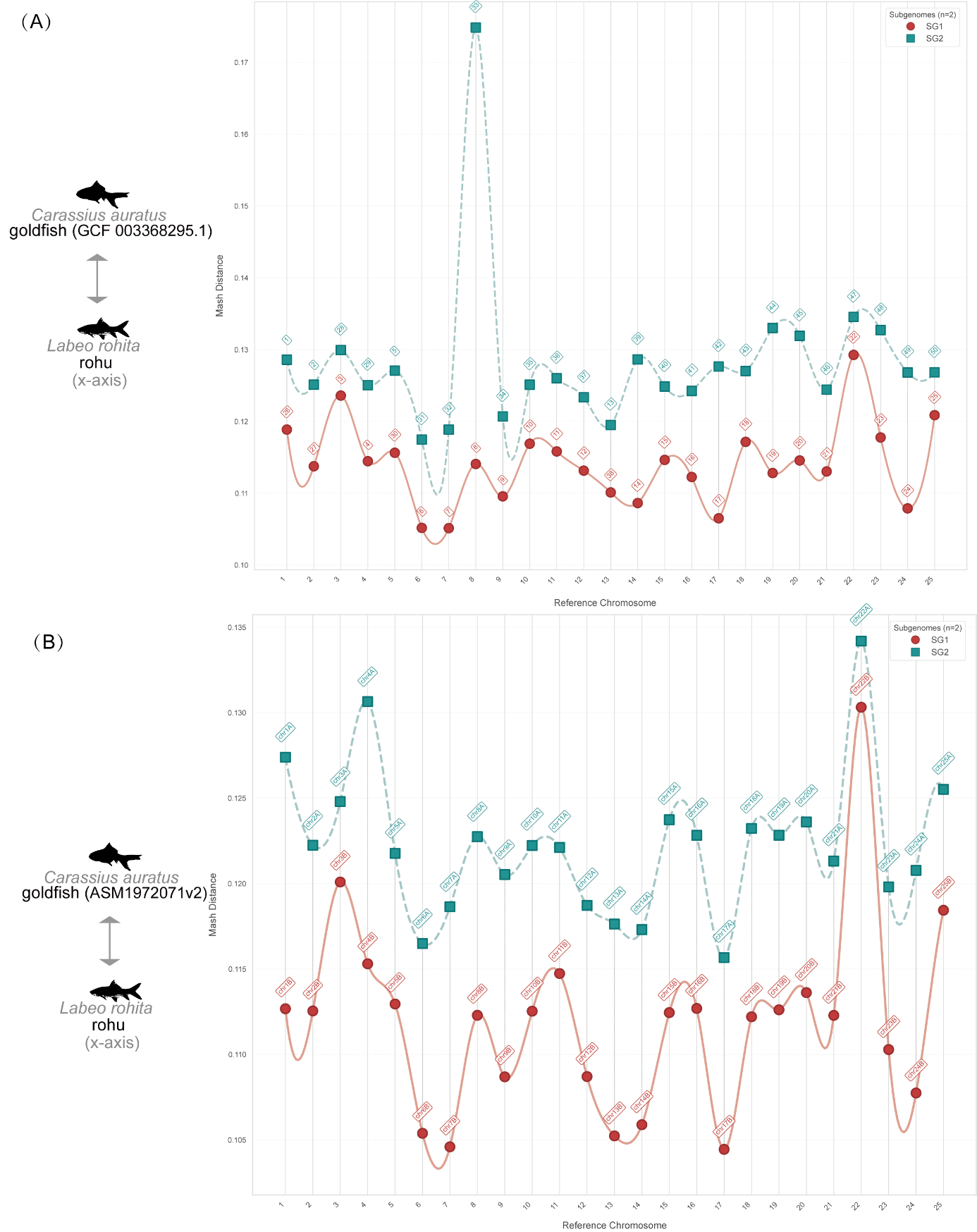


Figure S12. **Comparison of the Subgenome Partitioning Results Between Two Versions of the Goldfish Genome Identified Using the M3 Method with** Rohu **as the Reference**. The upper panel shows results based on genome version GCF_002268295.1, while the lower panel corresponds to ASM1972071v2.


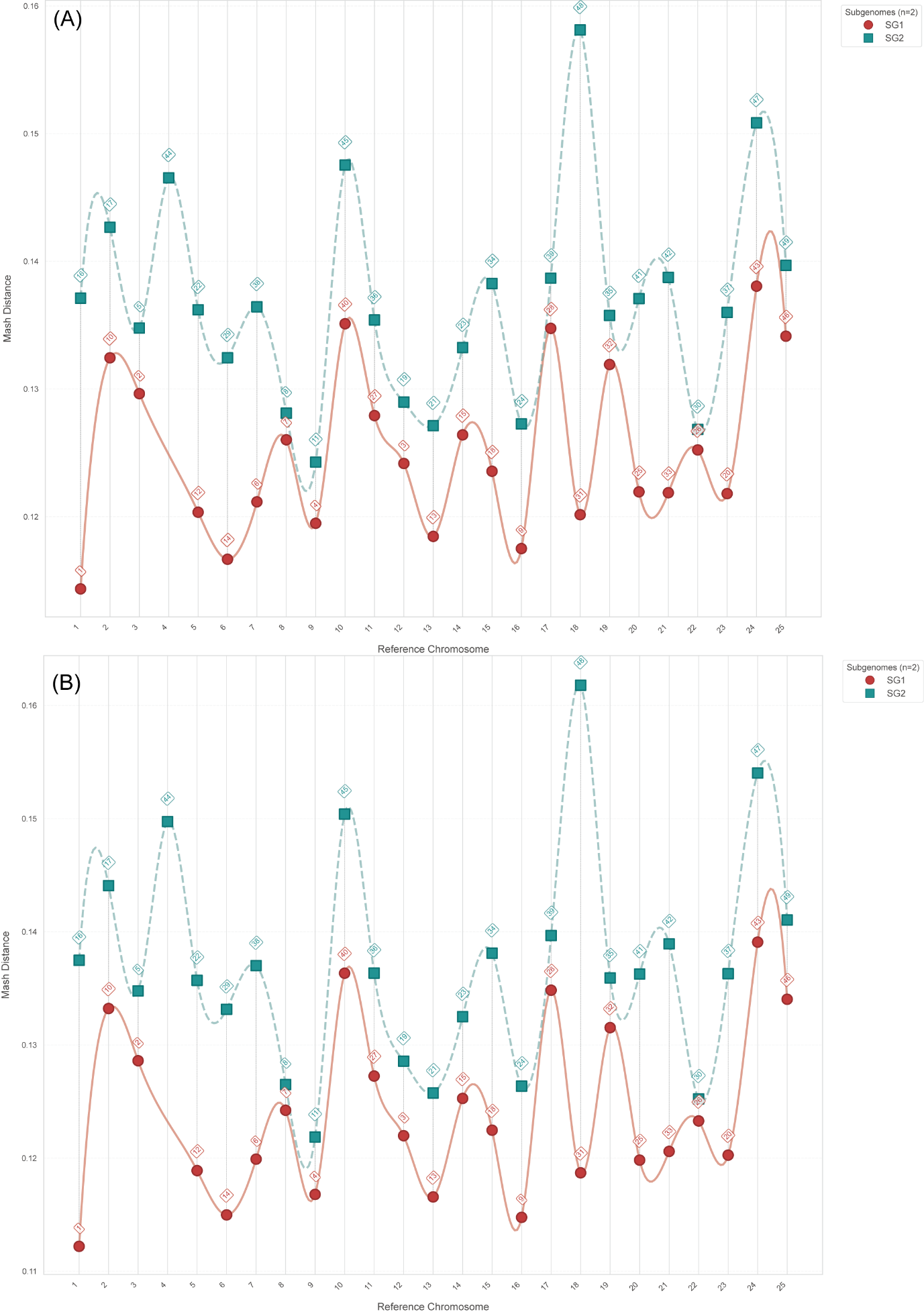


Figure S13. Subgenome Partitioning of the Golden Chinese Loach Identified Using the M3 Method. The upper panel shows results based on the original Golden Chinese Loach genome without repeat masking, while the lower panel displays results using the repeat-masked genome (repetitive sequences replaced with Ns), both of which are referred to as Yichang Sand Loach chromosomes.


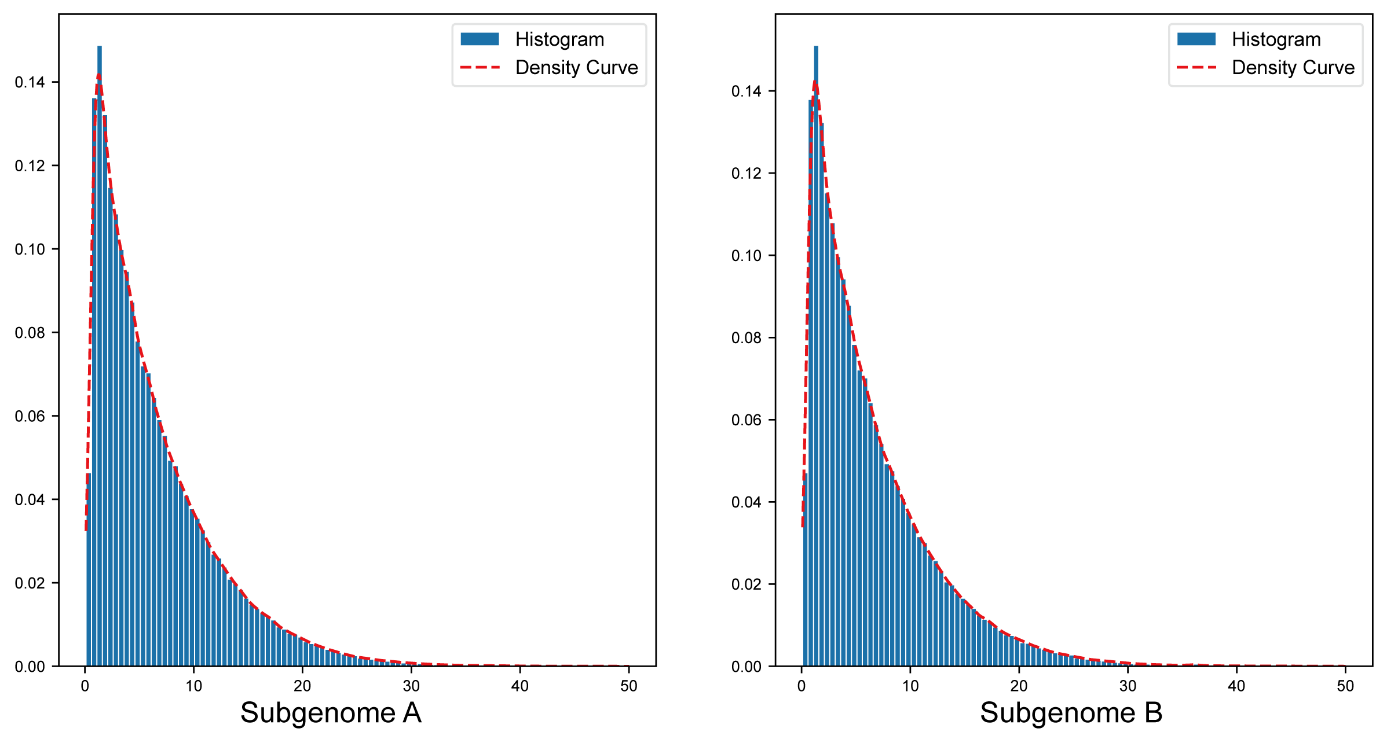


Figure S14. Distribution of Repeat Sequence Percent Divergence in the Wide-bodied Sand Loach


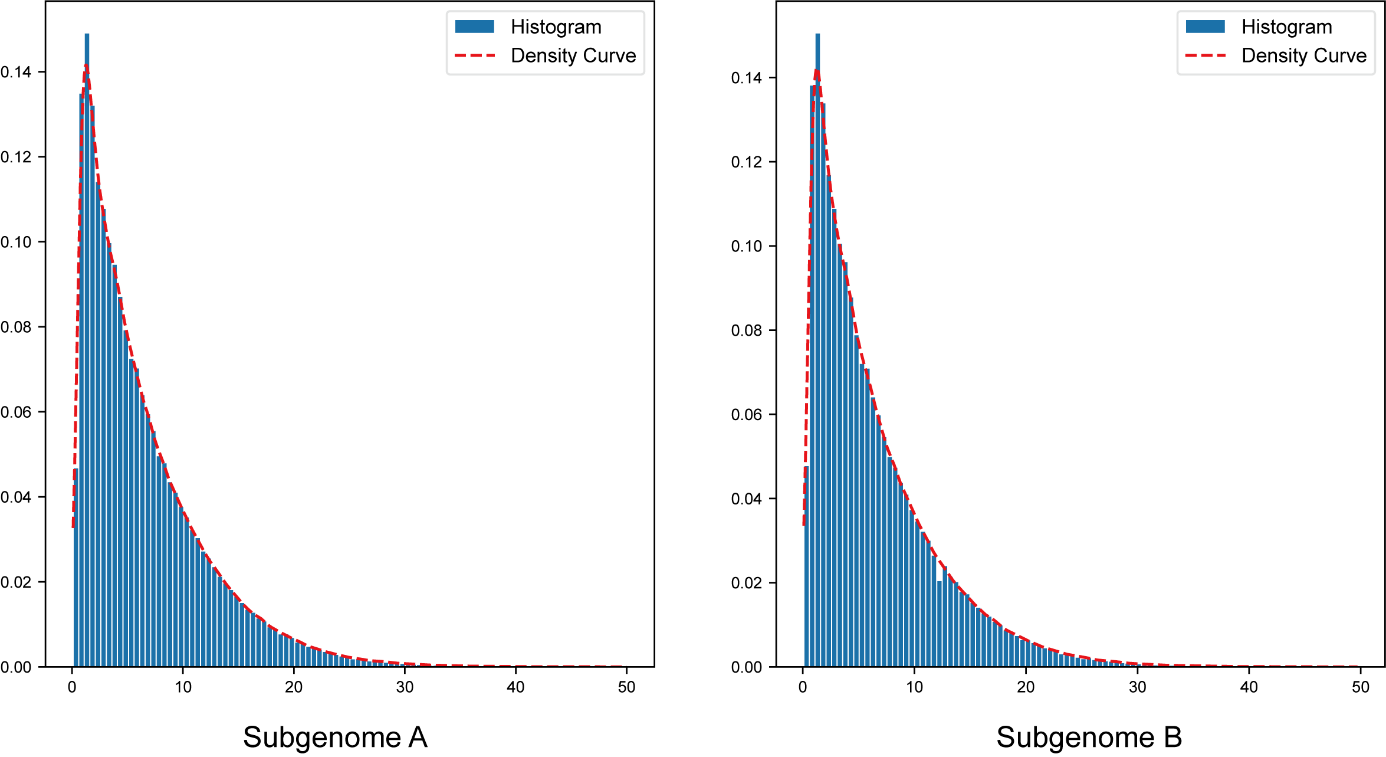


Figure S15. Distribution of Repeat Sequence Percent Divergence in the Golden Chinese Loach.


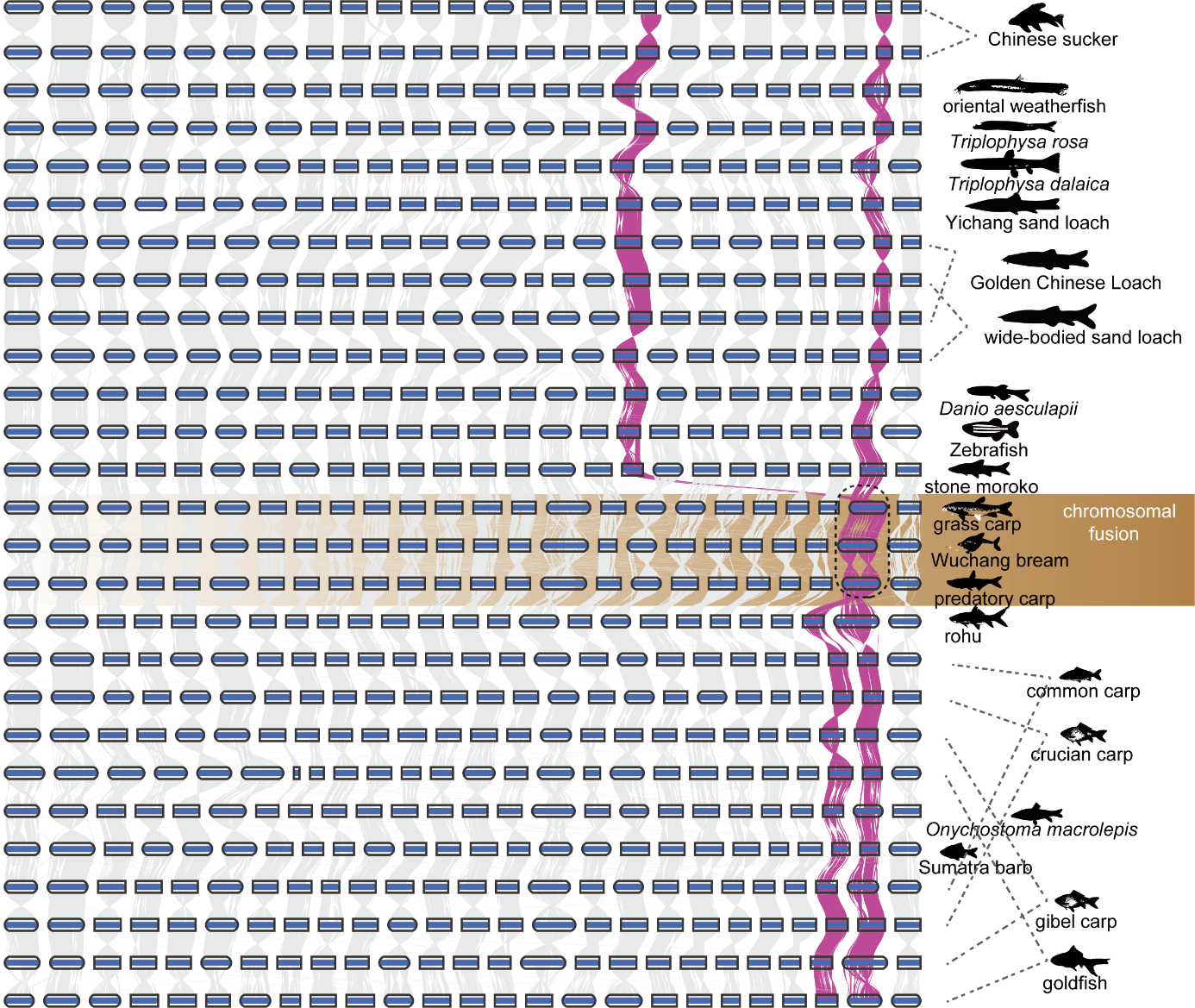


Figure S16. Chromosomal Rearrangement Comparison among Species in the Order Cypriniformes.

Table S1. Second-generation sequencing data statistics for the Golden Chinese Loach.

| file | format | type | num_seqs | sum_len | min_len | avg_len | max_len |
| --- | --- | --- | --- | --- | --- | --- | --- |
| E100033158_L01_71_1.fq.gz | FASTA | DNA | 206,238,812 | 30,935,821,800 | 150 | 150 | 150 |
| E100033158_L01_71_1.fq.gz | FASTA | DNA | 197,101,315 | 29,565,197,250 | 150 | 150 | 150 |

Table S2. Third-generation sequencing data statistics for the Golden Chinese Loach.

| file | format | type | num_seqs | sum_len | min_len | avg_len | max_len |
| --- | --- | --- | --- | --- | --- | --- | --- |
| 20211213-NPL3875-P6-PAH97912.pass.fa.gz | FASTA | DNA | 2,801,152 | 68,661,683,665 | 15 | 24,511.9 | 291,828 |

Table S3. Second-generation sequencing data statistics for the Yichang Sand Loach.

| file | format | type | num_seqs | sum_len | min_len | avg_len | max_len |
| --- | --- | --- | --- | --- | --- | --- | --- |
| HB-ILL1_1.clean.fq.gz | FASTQ | DNA | 203,428,620 | 30,514,293,000 | 150 | 150 | 150 |
| HB-ILL1_2.clean.fq.gz | FASTQ | DNA | 203,428,620 | 30,514,293,000 | 150 | 150 | 150 |

Table S4. Third-generation sequencing data statistics for the Yichang Sand Loach.

| file | format | type | num_seqs | sum_len | min_len | avg_len | max_len |
| --- | --- | --- | --- | --- | --- | --- | --- |
| HB.ONT.fastq.gz | FASTQ | DNA | 41,793,684 | 58,607,870,692 | 96 | 1,402.3 | 897,958 |

Table S5. Genome assembly results for the Golden Chinese Loach.

| Assembly level | scaffold (chromosome-level) | | contig | |
| --- | --- | --- | --- | --- |
|  | Length (bp) | Number | Length (bp) | Number |
| Maximum length | 25,079,155 |  | 24,364,878 |  |
| N10 | 19,461,878 | 4 | 18,411,358 | 4 |
| N20 | 18,743,000 | 8 | 15,847,482 | 9 |
| N30 | 17,516,996 | 13 | 15,352,556 | 14 |
| N40 | 16,227,500 | 18 | 13,477,309 | 20 |
| N50 | 15,539,000 | 23 | 12,906,393 | 26 |
| N60 | 14,930,258 | 28 | 10,726,322 | 33 |
| N70 | 14,081,812 | 34 | 7,844,000 | 42 |
| N80 | 13,403,342 | 39 | 5,852,476 | 53 |
| N90 | 8,901,000 | 47 | 1,013,229 | 85 |
| Total length | 807,262,951 |  | 807,113,951 |  |
| number>=0bp | 489 |  | 787 |  |
| Total length>=0bp | 807,262,951 |  | 807,113,951 |  |
| number>=2,000bp | 486 |  | 783 |  |
| Total length>=2,000bp | 807,259,074 |  | 807,110,002 |  |
| number>=10,000bp | 467 |  | 759 |  |
| Total length>=10,000bp | 807,145,137 |  | 806,974,588 |  |
| GC rate | 0.394 |  | 0.394 |  |

Table S6. Genome assembly results for the Yichang Sand Loach.

| Assembly level | scaffold (chromosome-level) | | contig | |
| --- | --- | --- | --- | --- |
|  | Length (bp) | Number | Length (bp) | Number |
| Maximum length | 30,460,764 |  | 18,696,162 |  |
| N10 | 27,650,186 | 3 | 14,754,454 | 4 |
| N20 | 25,731,012 | 5 | 10,235,865 | 10 |
| N30 | 24,185,245 | 7 | 7,745,260 | 17 |
| N40 | 22,871,451 | 10 | 6,165,984 | 26 |
| N50 | 22,300,933 | 13 | 4,253,505 | 38 |
| N60 | 21,364,690 | 16 | 2,539,530 | 56 |
| N70 | 21,128,513 | 18 | 1,466,813 | 88 |
| N80 | 19,622,984 | 22 | 843,250 | 142 |
| N90 | 16,677,440 | 25 | 184,399 | 294 |
| Total length | 614,141,018 |  | 614,100,318 |  |
| number>=0bp | 1,429 |  | 1,836 |  |
| Total length>=0bp | 614,141,018 |  | 614,100,318 |  |
| number>=2,000bp | 1,429 |  | 1,836 |  |
| Total length>=2,000bp | 614,141,018 |  | 614,100,318 |  |
| number>=10,000bp | 1,092 |  | 1,499 |  |
| Total length>=10,000bp | 612,217,327 |  | 612,176,627 |  |
| GC rate | 0.394 |  | 0.394 |  |

Table S7. BUSCO-based genome completeness estimates for the Golden Chinese Loach (actinopterygii_odb10).

|  | Number | Percent |
| --- | --- | --- |
| Complete BUSCOs (C) | 3,552 | 97.60% |
| Complete and single-copy BUSCOs (S) | 2,402 | 66.00% |
| Complete and duplicated BUSCOs (D) | 1,150 | 31.60% |
| Fragmented BUSCOs (F) | 12 | 0.30% |
| Missing BUSCOs (M) | 76 | 2.10% |
| Total BUSCO groups searched | 3,640 | 100% |

Table S8. BUSCO-based genome completeness estimates for the Yichang Sand Loach (actinopterygii_odb10).

|  | Number | Percent |
| --- | --- | --- |
| Complete BUSCOs (C) | 3,556 | 97.60% |
| Complete and single-copy BUSCOs (S) | 3,496 | 96.00% |
| Complete and duplicated BUSCOs (D) | 60 | 1.60% |
| Fragmented BUSCOs (F) | 23 | 0.60% |
| Missing BUSCOs (M) | 61 | 1.80% |
| Total BUSCO groups searched | 3,640 | 100% |

Table S9. Repetitive sequence statistics for the Golden Chinese Loach genome.

|  | number of elements | length occupied （bp） | percentage |
| --- | --- | --- | --- |
| Retroelements | 363,101 | 86,621,956 | 10.55% |
| SINEs: | 48,479 | 3,669,342 | 0.45% |
| Penelope | 1,095 | 337,906 | 0.04% |
| LINEs: | 181,711 | 3,8128,330 | 4.65% |
| CRE/SLACS | 0 | 0 | 0.00% |
| L2/CR1/Rex | 111,434 | 23,530,077 | 2.87% |
| R1/LOA/Jockey | 2,506 | 534,539 | 0.07% |
| R2/R4/NeSL | 617 | 173,323 | 0.02% |
| RTE/Bov-B | 28,437 | 6,915,399 | 0.84% |
| L1/CIN4 | 6,578 | 1,291,527 | 0.16% |
| LTR elements: | 132,911 | 44,824,284 | 5.46% |
| BEL/Pao | 8,660 | 1,203,226 | 0.15% |
| Ty1/Copia | 844 | 125,334 | 0.02% |
| Gypsy/DIRS1 | 90,484 | 3,7658,739 | 4.59% |
| Retroviral | 15,079 | 4,256,600 | 0.52% |
| DNA transposons | 849,847 | 75,090,890 | 9.15% |
| hobo-Activator | 254,054 | 18,826,127 | 2.29% |
| Tc1-IS630-Pogo | 156,490 | 14,903,068 | 1.82% |
| En-Spm | 0 | 0 | 0.00% |
| MuDR-IS905 | 0 | 0 | 0.00% |
| PiggyBac | 17,129 | 1,187,411 | 0.14% |
| Tourist/Harbinger | 97,281 | 10,224,348 | 1.25% |

Table S10. Repetitive sequence statistics for the Yichang Sand Loach genome.

|  | number of elements | length occupied （bp） | percentage |
| --- | --- | --- | --- |
| Retroelements | 213,875 | 49,051,843 | 7.99 % |
| SINEs: | 27,781 | 2,266,486 | 0.37 % |
| Penelope | 1,137 | 687,128 | 0.11 % |
| LINEs: | 112,489 | 22,596,909 | 3.68 % |
| CRE/SLACS | 0 | 0 | 0.00 % |
| L2/CR1/Rex | 68,014 | 13,650,512 | 2.22 % |
| R1/LOA/Jockey | 2,315 | 549,249 | 0.09 % |
| R2/R4/NeSL | 562 | 211,808 | 0.03 % |
| RTE/Bov-B | 13,783 | 1,845,855 | 0.30 % |
| L1/CIN4 | 4,945 | 1,137,996 | 0.19 % |
| LTR elements: | 73,605 | 24,188,448 | 3.94 % |
| BEL/Pao | 7,429 | 2,328,757 | 0.38 % |
| Ty1/Copia | 624 | 300,538 | 0.05 % |
| Gypsy/DIRS1 | 43,658 | 17,185,158 | 2.80 % |
| Retroviral | 9,585 | 2,838,745 | 0.46 % |
| DNA transposons | 608,923 | 64,622,601 | 10.52 % |
| hobo-Activator | 187,855 | 16,854,089 | 2.74 % |
| Tc1-IS630-Pogo | 111,596 | 13,882,242 | 2.26 % |
| En-Spm | 0 | 0 | 0.00 % |
| MuDR-IS905 | 0 | 0 | 0.00 % |
| PiggyBac | 9,335 | 781,491 | 0.13 % |
| Rolling-circles | 50,211 | 9,380,976 | 1.53 % |
| Unclassified: | 407,139 | 119,220,439 | 19.41 % |
| Total interspersed repeats: |  | 232,894,883 | 37.92 % |
| Small RNA: | 0 | 0 | 0.00 % |
| Satellites: | 11,516 | 3,309,853 | 0.54 % |

Table S11. BUSCO-based annotation completeness for the Golden Chinese Loach genome (actinopterygii_odb10).

|  | Number | Percent |
| --- | --- | --- |
| Complete BUSCOs (C) | 3,177 | 94.7% |
| Complete and single-copy BUSCOs (S) | 2,402 | 61.7% |
| Complete and duplicated BUSCOs (D) | 2,071 | 33.0% |
| Fragmented BUSCOs (F) | 72 | 2.1%, |
| Missing BUSCOs (M) | 105 | 3.2% |
| Total BUSCO groups searched | 3,640 | 100% |

Table S12. BUSCO-based annotation completeness for the Yichang Sand Loach genome (actinopterygii_odb10).

|  | Number | Percent |
| --- | --- | --- |
| Complete BUSCOs (C) | 3,334 | 91.6% |
| Complete and single-copy BUSCOs (S) | 3,271 | 89.9% |
| Complete and duplicated BUSCOs (D) | 63 | 1.7% |
| Fragmented BUSCOs (F) | 112 | 3.1% |
| Missing BUSCOs (M) | 194 | 5.3% |
| Total BUSCO groups searched | 3,640 | 100% |

Table S13. Species included in the comparative genomic analysis and their sources.

| Species name | groups | common name | Order | Accession number or study |
| --- | --- | --- | --- | --- |
| *Ictalurus furcatus* | outgroup | blue catfish | Siluriforme | GCA 023375685.2 |
| *Danio rerio* | ingroup | zebrafish | Cypriniforme | GCA 000002035.4 |
| *Carassius gibelio* | ingroup | gibel carp | Cypriniforme | GCF 023724105.1 |
| *Cyprinus carpio* | ingroup | common carp | Cypriniforme | GCF 018340385.1 |
| *Ctenopharyngodon idella* | ingroup | grass carp | Cypriniforme | GCF 019924925.1 |
| *Carassius auratus* | ingroup | goldfish | Cypriniforme | GCF 003368295.1 |
| *Carassius auratus* | / | goldfish | Cypriniforme | ASM1972071v2 |
| *Megalobrama amblycephala* | ingroup | Wuchang bream | Cypriniforme | GCF 018812025.1 |
| *Labeo rohita* | ingroup | rohu | Cypriniforme | GCF 022985175.1 |
| *Misgurnus anguillicaudatus* | ingroup | oriental weatherfish | Cypriniforme | GCF 027580225.1 |
| *Carassius* | ingroup | crucian carp | Cypriniforme | GCF 963082965.1 |
| *Myxocyprinus asiaticus* | ingroup | Chinese sucker | Cypriniforme | GCF 019703515.2 |
| *Onychostoma macrolepis* | ingroup | Onychostoma macrolepis | Cypriniforme | GCF 012432095.1 |
| *Pseudorasbora parva* | ingroup | stone moroko | Cypriniforme | GCF 024679245.1 |
| *Triplophysa dalaica* | ingroup | Triplophysa dalaica | Cypriniforme | GCF 015846415.1 |
| *Puntigrus tetrazona* | ingroup | Sumatra barb | Cypriniforme | GCF 018831695.1 |
| *Chanodichthys erythropterus* | ingroup | predatory carp | Cypriniforme | GCF 024489055.1 |
| *Danio aesculapii* | ingroup | Danio aesculapii | Cypriniforme | GCF 903798145.1 |
| *Triplophysa rosa* | ingroup | Triplophysa rosa | Cypriniforme | GCF 024868665.1 |
| *Sinibotia reevesae* | ingroup | wide-bodied sand loach | Cypriniforme | [a] |
| *Parabotia fasciatus* | ingroup | Yichang sand loach | Cypriniforme | This study |
| *Sinibotia superciliaris* | ingroup | Golden Chinese Loach | Cypriniforme | This study |

[a] Lv, Yunyun, Yanping Li, Yu Huang, Jun Wang, Zhilin Tian, Yang He, Jinrong Shi et al. “Deciphering genome-wide molecular pathways for exogenous *Aeromonas hydrophila* infection in wide-bodied sand loach (*Sinibotia reevesae*).” Aquaculture Reports 35 (2024): 102033.
